# Cost-Effectiveness and Harm-Benefit Analyses of Risk-Based Screening Strategies for Breast Cancer

**DOI:** 10.1371/journal.pone.0086858

**Published:** 2014-02-03

**Authors:** Ester Vilaprinyo, Carles Forné, Misericordia Carles, Maria Sala, Roger Pla, Xavier Castells, Laia Domingo, Montserrat Rue, the Interval Cancer (INCA) Study Group

**Affiliations:** 1 Basic Medical Sciences Department, Biomedical Research Institut of Lleida (IRBLLEIDA), Lleida, Catalonia, Spain; 2 Basic Medical Sciences Department, University of Lleida, Lleida, Catalonia, Spain; 3 Economics Department and Research Centre on Industrial and Public Economics (CREIP), Rovira i Virgili University, Reus, Catalonia, Spain; 4 Department of Epidemiology and Evaluation, Institut Municipal d'Investigació Mèdica-Parc de Salut Mar, Mar Teaching Hospital, Barcelona, Catalonia, Spain; 5 Health Services Research Network in Chronic Diseases (REDISSEC), Spain; 6 Surgery Department, Rovira i Virgili University, Reus, Catalonia, Spain; 7 General and Digestive Surgery Department, Joan XXIII Teaching Hospital, Tarragona, Catalonia, Spain; University of Torino, Italy

## Abstract

The one-size-fits-all paradigm in organized screening of breast cancer is shifting towards a personalized approach. The present study has two objectives: 1) To perform an economic evaluation and to assess the harm-benefit ratios of screening strategies that vary in their intensity and interval ages based on breast cancer risk; and 2) To estimate the gain in terms of cost and harm reductions using risk-based screening with respect to the usual practice. We used a probabilistic model and input data from Spanish population registries and screening programs, as well as from clinical studies, to estimate the benefit, harm, and costs over time of 2,624 screening strategies, uniform or risk-based. We defined four risk groups, low, moderate-low, moderate-high and high, based on breast density, family history of breast cancer and personal history of breast biopsy. The risk-based strategies were obtained combining the exam periodicity (annual, biennial, triennial and quinquennial), the starting ages (40, 45 and 50 years) and the ending ages (69 and 74 years) in the four risk groups. Incremental cost-effectiveness and harm-benefit ratios were used to select the optimal strategies. Compared to risk-based strategies, the uniform ones result in a much lower benefit for a specific cost. Reductions close to 10% in costs and higher than 20% in false-positive results and overdiagnosed cases were obtained for risk-based strategies. Optimal screening is characterized by quinquennial or triennial periodicities for the low or moderate risk-groups and annual periodicity for the high-risk group. Risk-based strategies can reduce harm and costs. It is necessary to develop accurate measures of individual risk and to work on how to implement risk-based screening strategies.

## Introduction

Early detection of breast cancer (BC) reduces mortality and may improve quality of life for most of the women diagnosed early by mammographic exams [1]. Nevertheless, screening healthy women is expensive and may cause harms (e.g. false positive results, overdiagnosis) in many of them [2]–[5]. In order for organized screening programs to be justified in this time of economic constraints, overall benefits should outweigh harms at a reasonable cost. Moreover, an economic evaluation is especially necessary when screening is funded by community resources.

Organized screening programs for early detection of BC provide screening services where all eligible women are treated as equal risk. For instance, the European guidelines recommend offering mammography screening to women aged 50–69 every two years [6]. This one-size-fits-all or uniform paradigm is starting to shift toward personalizing screening strategies based on breast cancer risk. In 2005 the Institute of Medicine (IOM) identified that personalized screening was crucial to improving the early detection of breast cancer [7]. More recently, Schousboe *et al.* [8], using a Markov microsimulation model, found that the cost-effectiveness of screening mammography depended on a woman's age, breast density, family history, and history of breast biopsy. Based on their results, mammography every two years was cost-effective for women aged 40 to 49 years with relatively high breast density or additional risk factors for breast cancer. And mammography every three to four years was cost-effective for women aged 50 to 79 years with low breast density and no other risk factors. van Ravesteyn *et al.* [9], using different microsimulation models, determined that women aged 40 to 49 years with a twofold increase in risk have similar harm-benefit ratios for biennial screening mammography as average-risk women aged 50 to 74 years.

In a previous study, we performed an economic evaluation of uniform screening strategies that had different periodicities and varied in the ages at starting or ending the screening exams [10]. The present study has two objectives that extend our previous work: 1) To perform an economic evaluation and to assess the harm-benefit ratios of screening strategies that vary in their intensity and interval ages based on BC risk; and 2) To estimate the gain in terms of cost and harm reductions using risk-based screening with respect to the usual practice.

## Methods

### The model and model inputs

We used the probabilistic model developed by Lee and Zelen (LZ), which has been described elsewhere [11]–[13]. Further details of the model can be found in Appendix S1, section A. The model assumes a four-state progressive disease with 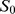: disease-free state, 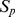: preclinical state (asymptomatic disease that can be diagnosed by a special exam), 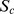: clinical state (diagnosis by symptomatic detection), and 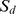: death from BC. The LZ model consists of a set of equations that allow to estimate the cumulative probability of death for a particular cohort exposed to a specific screening scenario or to no screening, after T years of follow-up. Since the model is analytical, for each specific set of inputs, the model run produces the same results. The model also provides incidence and prevalence of BC over time, both measures necessary for the estimation of treatment and follow-up costs.

The model requires input data that was obtained from different sources. BC incidence and survival, and mortality from other causes refer to cohorts born in Catalonia (Spain) in the period 1948–1952 [10], [14]–[17]. The sojourn time in the pre-clinical state, the distribution of stages at diagnosis (Table S1 in Appendix S1) and the sensitivity of mammography were obtained by Lee and Zelen from published randomized clinical trials and observational studies [12]. Based on previous work of Zelen and Feinleib [18] and Day and Walter [19] on the randomized clinical trial of the Health Insurance Plan (HIP), it was assumed that the preclinical sojourn time follows an exponential distribution with an age dependent mean equal to 2 years for age<40 and 4 years for age>50. In the (40–50] age interval the mean sojourn time increases linearly from 2 to 4 years. The additional inputs are described below in the next subsections. All the calculations assumed an initial population of 100,000 women at birth. The time horizon for the study was 40–79 years of age.

The research protocol was approved by the institutional review board and ethics committee of the Hospital Universitari Arnau de Vilanova de Lleida (Spain) which waived the need for informed consent.

### Risk of invasive breast cancer

We started estimating the age-specific risk of invasive BC for our study cohort, using the model published elsewhere by Martinez-Alonso *et al.* [17]. Details of the model can be found in Appendix S1, Section B.1. Then, following Tice *et al.* [20] and Schousboe *et al.* [8], age-specific BC risk groups were defined according to the following variables: breast density (measured using the Breast Imaging Report and Database System (BI-RADS) categories 1 to 4 [21]), family history of BC in first degree relatives (yes/no) and personal history of breast biopsy (yes/no). Details can be found in Appendix S1, section B.2.

We obtained four aggregated risk groups that combined the profiles of women that had similar levels of BC incidence over time: 1) Low (L) risk which included Category 1 breast density with at most one risk factor - family history or breast biopsy - and Category 2 breast density with no risk factors; 2) Medium-Low (ML) risk which included Category 1 breast density with two risk factors, Category 2 breast density with one risk factor, and Categories 3 or 4 breast density with no risk factors; 3) Medium-High (MH) risk which included Category 2 breast density with two risk factors, Categories 3 or 4 breast density with one risk factor; and 4) High (H) risk which included Categories 3 or 4 breast density with two risk factors. The frequency distributions of the risk groups was 39.6%, 42.8%, 15.6% and 2.0% for L, ML, MH and H, respectively.

The incidence rates of the four aggregated risk groups were estimated as weighted sums of detailed incidence curves (see Section B.2, Tables S2, S3, and Figure S1 in Appendix S1). The weights were based on the prevalences of each combination of risk factors obtained from the Risk Estimation Dataset of the Breast Cancer Surveillance Consortium (BCSC) [22].

### The screening strategies

We analyzed 2,625 screening strategies, 24 of them uniform and 2,601 risk-based. The risk-based strategies were obtained combining the exam periodicity (annual (A), biennial (B), triennial (T), and quinquennial (Q, [every five years])), the starting ages (40, 45 and 50 years) and the ending ages (69 and 74 years) in the four risk groups, L, ML, MH and H. In the following sections, uniform strategies are abbreviated as B5069 or B4574, for biennial exams in the 50–69 or in the 45–74 age groups, respectively. Risk-based strategies are abbreviated with four strings, e.g. Q5069-Q4574-T4574-A4074, that correspond to the L, ML, MH and H risk groups, respectively. A sample of the studied screening strategies is presented in Table S4 in Appendix S1.

### The benefits

For each screening strategy and for the background, we measured the benefit of screening with two outcomes: the number of lives extended, LE, and the number of quality-adjusted life years gained, QALY. Because of the lack of Spanish data, the QALYs were estimated using the work of Lidgren *et al.* [23] in a sample of 361 Swedish women with localized, recurrent, or metastatic breast cancer (See Table S5 in Appendix S1). We considered the Lidgren's study more robust and suitable than other studies that used expert opinion or healthy population to obtain quality of life estimates associated with breast cancer. We used the values obtained from the EuroQol EQ-5D in the Lidgren's study. For women that did not die of BC we considered a loss of QALYs in the first five years following the diagnosis. For women that died of breast cancer, we considered that the last four years of their lives or the time from diagnosis to death, if they lived less than four years, were spent in a metastatic stage, independently of the stage at diagnosis. See section C in Appendix S1 for further details.

### The harms

#### False positive (FP) results

We used the FP rates for non-invasive and invasive tests obtained from the Cumulative Risk of False Positive Study (RAFP) study which included 74 distinct radiology units in eight regions of Spain, from March 1990 to December 2006 [2]. The RAFP study included 1,565,364 women that underwent 4,739,498 mammographic exams. The FP rates were age and exam specific. We multiplied the FP rates by the number of women at risk for BC, at each specific exam, to estimate the number of women that would receive additional non-invasive (e.g. ultrasound) or invasive tests (e.g. biopsy). See further details in Appendix S1, section D and Tables S6 and S7.

#### Interval cancers and false-negative (FN) results

In our model, true interval tumors correspond to those that appear between exams and were not in the pre-clinical state when the previous exam was performed. FN cases are tumors that were not detected in the previous exams due to lack of sensitivity of the screening test. We considered that all tumors in pre-clinical state in the previous exam were FN.

#### Overdiagnosis

Screening may cause overdiagnosis when it detects tumors which would never have been diagnosed during a lifetime without screening because of the lack of progressive potential or death from other causes. To estimate overdiagnosis we made some additional assumptions. We differentiated between overdiagnosis of invasive BC and ductal carcinoma in situ (DCIS). For both types of tumors we assumed that: 1) overdiagnosis only happens when a mammographic exam is performed, 2) a woman with an overdiagnosed tumor would not die of breast cancer, and 3) QALYs and costs of treatment (initial and follow-up) for women with overdiagnosed tumors are the same as for Stage I BC.

#### Overdiagnosis of invasive breast cancer

Estimates of overdiagnosis show high variation depending on the study design and the method used [3], [4], [17], [24]–[28]. Based on the reported data, an overdiagnosis rate of 15% can be considered a sensible value.

Using the incidence model described in Appendix S1, section B.1 [17], and taking into account the distribution of the sojourn times in the preclinical state and the sensitivity of mammography (as in the LZ model), we estimated the number of BC cases that would be detected in the screening exams. Then, for each screening strategy, we assumed an overdiagnosis rate of 15% in the mammography exams. This assumption makes it possible to associate overdiagnosis with mammography exams, in the sense that more intensive screening strategies are considered to produce a higher overdiagnosis burden. For any screening strategy, an overdiagnosis estimate of 15% of the screen-detected cases gives an overall estimate lower than 15%, depending on the distribution of exam-detected and interval cases. For further details about how the overdiagnosis rate has been applied see section E and Table S8 in Appendix S1.

#### DCIS attributable to screening

To estimate the impact of screening on detection of DCIS we obtained the incidence and Census data from the Girona and Tarragona Cancer Registries in the period 1983–2008. Data on mammography use was obtained, for the Girona and Tarragona provinces, from three health surveys performed in the years 1994, 2002 and 2006 [29], [30]. Section F in Appendix S1 explains in detail the analysis conducted to estimate the excess of DCIS attributable to sceening. From this analysis we estimated an excess of 31.13 DCIS cases per 100,000 mammograms, with respect to a strategy of no screening (Table S9 and Figures S2 and S3 in Appendix S1).

Because DCIS is treated when detected, it is not possible to accurately estimate the fraction of detected DCIS that would progress to invasive disease. A review of the literature showed that between 14% and 53% of DCIS may progress to invasive cancer over a period of 10 or more years [31]. In our study we have assumed that 1/3 of the DCIS detected by mammography would progress to invasive cancer. With this assumption, the estimated excess number of DCIS attributable to screening was approximately 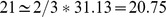 per 100,000 mammograms, or 0.21 per 1,000 mammograms. In the sensitivity analysis we have estimated the proportion of DCIS that progress to be equal to 2/3 or to 1/6 of 31.13 per 100,000 mammograms.

### Costs

We have adopted the perspective of the national health system and considered only direct healthcare costs. We have partitioned the estimation of costs into four parts: screening and diagnosis confirmation, initial treatment, follow-up and advanced care costs. All costs were valued in 2012 euros and both costs and outcomes have been discounted at an annual rate of 3%, according to the economic evaluation guidelines of the Spanish Ministry of Health [32].

The costs of screening mammograms, complementary tests and administrative expenses were obtained from the Early Detection Program of *Parc de Salut Mar* (PSMAR) in the city of Barcelona. Data on treatment costs were obtained from a database that included 592 women consecutively diagnosed and initially treated for BC at the PSMAR in Barcelona in the period January 1st, 2000–December 31, 2003 [10].

### Cost-effectiveness and harm-benefit analyses

To compare the relative costs and outcomes of the different strategies, we calculated the incremental cost-effectiveness ratio (ICER). The ICER is defined as the ratio of the change in costs to the change in effects of a specific intervention compared to an alternative. The ICER indicates the additional cost of obtaining one additional unit of outcome. We obtained the cost-effectiveness frontier, also called the Pareto frontier, which contains the efficient alternatives for which no alternative policy exists that results in better effects for lower costs.

To perform a harm-benefit analyses, we ordered the studied strategies from less to more adverse effects and obtained the incremental harm-benefit ratio of each strategy in relation to the previous one. We also obtained the harm-benefit frontier.

### Selection of optimal strategies

To search for optimal strategies taking into account benefit, costs and harms, we selected the most recommended uniform strategy in Europe, biennial exams in the 50–69 age interval (B5069), or the alternative towards which some countries are moving, biennial exams in the 45–74 age interval (B4574), as reference strategies. Then, for each reference strategy we obtained the intersection of the subsets that contained strategies with similar benefit (between 1 and 1.05 times) than the reference strategy and lower cost and harms in terms of FP results and overdiagnosed cases (invasive and DCIS). The resulting strategies were located at or near the cost-effectiveness and harm-benefit frontiers with values in the x-axis near the B5069 or B4574 benefit values. We did not include the FN results in the intersection but we assessed them in the resulting optimal subset.

### Validation of the model

We have compared our results with the results of three published reviews, the Cochrane systematic review [33], the Independent UK Panel on Breast Cancer Screening review [34], and the Euroscreen comprehensive review of European screening programs [35]. In addition, we have checked the results of the INterval CAncer (INCA) study in Spain, which included 645,764 women aged 45/50 to 69 years that participated biennially in seven population-based screening programs, from January 2000 to December 2006 (not yet published). A total of 1,508,584 mammograms were included in the study. The cohort was followed until June 2009 for breast cancer identification, resulting in 5,311 cases screen-diagnosed and 1,682 interval cancers.

We have compared the following summary indicators in the INCA study and the uniform B4569 strategy of our model: 1) frequencies of screen-detected and interval cancer, by age-group, 2) sensitivity of the program defined as the ratio of the number of tumors detected in the screening exams between all the detected tumors, 3) distribution of true interval cases and FN, by time since last mammogram, and 4) distribution of stages at diagnosis, by type of detection (screening or symptomatic).

### Sensitivity analysis

There is uncertainty associated with the model inputs and there is also uncertainty associated with the model structure. It is complex and computationally intensive to obtain the variance of the model estimates. Instead, we performed univariate sensitivity analyses to study the impact on our conclusions when some of the inputs were modified. First, we changed the four risk group distributions assuming that 20% of women in the L, ML, and MH groups migrated to the next higher risk group. The new risk group distributions was 31.7%, 42.1%, 21.1% and 5.1%, for L, ML, MH and H, respectively. Second, we changed the amount of overdiagnosis of invasive tumors to 0%, 5% and 25%. Third, we changed the excess of DCIS to 0.1 and 0.26 per 1,000 mammograms. Fourth, we tested the effect of changing the costs of cancer treatment to two-fold and five-fold the costs of the main analysis. Fifth, we assessed the effect of changes in the disutility by false-positive result on QALY. We used zero and two times the disutility of the main analysis.

### Data availability

All the input data will be available to researchers upon request.

## Results

### Cost-effectiveness and harm-benefit analyses

**Figure 1:**
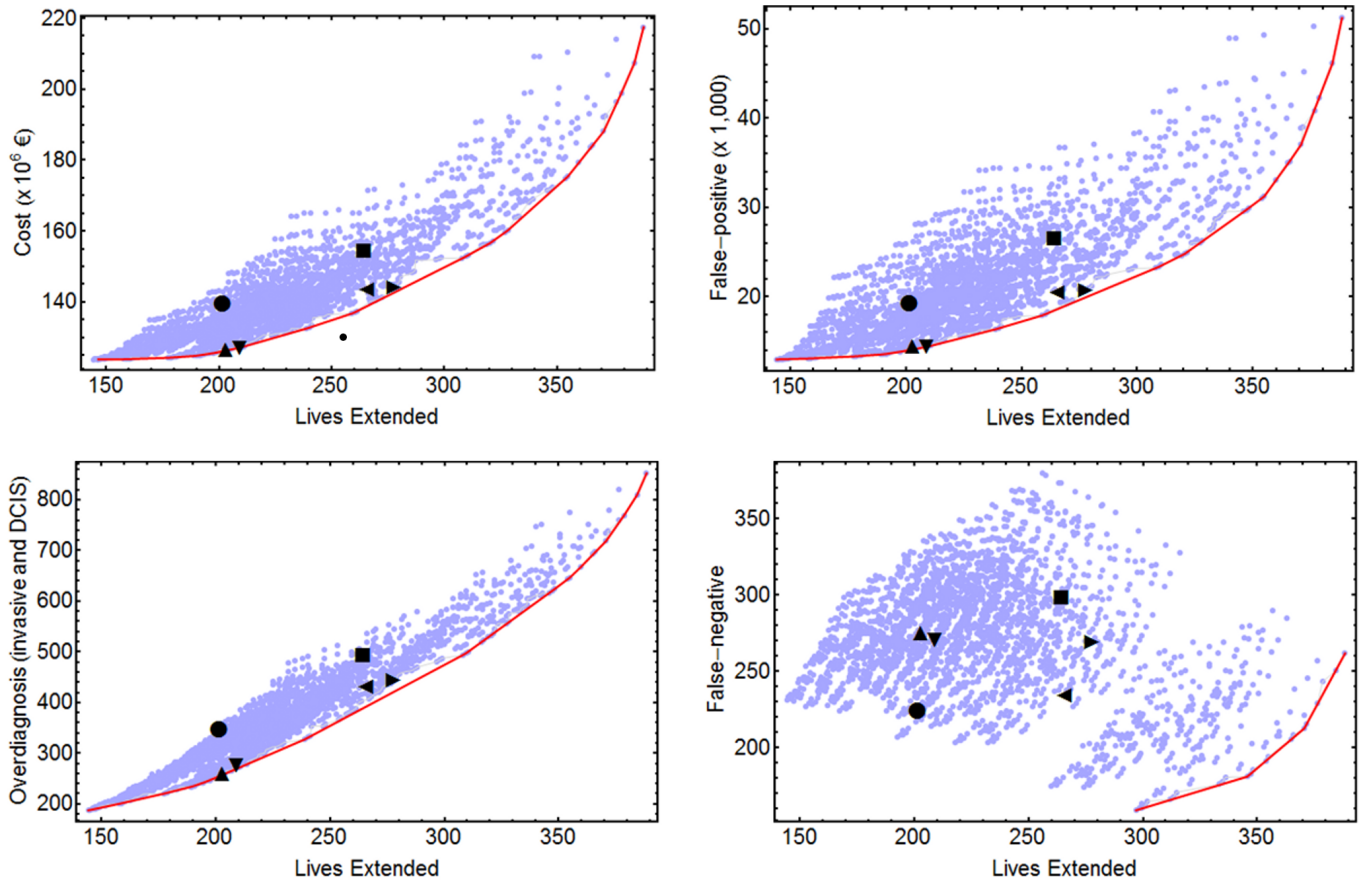
Cost-effectiveness and harm-benefit analyses for 2,625 early detection strategies. Effect measured in lives extended. Dots represent specific screening strategies. Results obtained with an annual discount of 3%. •: uniform B5069; ▪: uniform B4574. ▴: risk-based Q5074-Q5074-Q4574-A4574; ▾: risk-based Q5074-Q5074-T5074-A5074. ◂: risk-based T5069-B5074-A5074-A5074; ▸: risk-based T5074-T5074-A4574-A4574. Exams periodicities: A = annual, B = biennial, T = triennial, Q = quinquennial. The first two numbers refer to the age at starting the exams and the last two numbers refer to the age at the last exam. In the risk-based strategies, the four strings correspond to the Low, Medium-Low, Medium-High and High risk groups, respectively.

Benefits, harms, and costs of each screening strategy were obtained as a function of the risk-groups' incidence and the screening characteristics (periodicity and age-interval of exams by risk group). Figures 1 and 2 contain an overview of benefits, harms, and costs of all 2,625 strategies evaluated. The strategies that gave the best value for money can be found in Tables S10 and S11 in Appendix S1.

**Figure:**
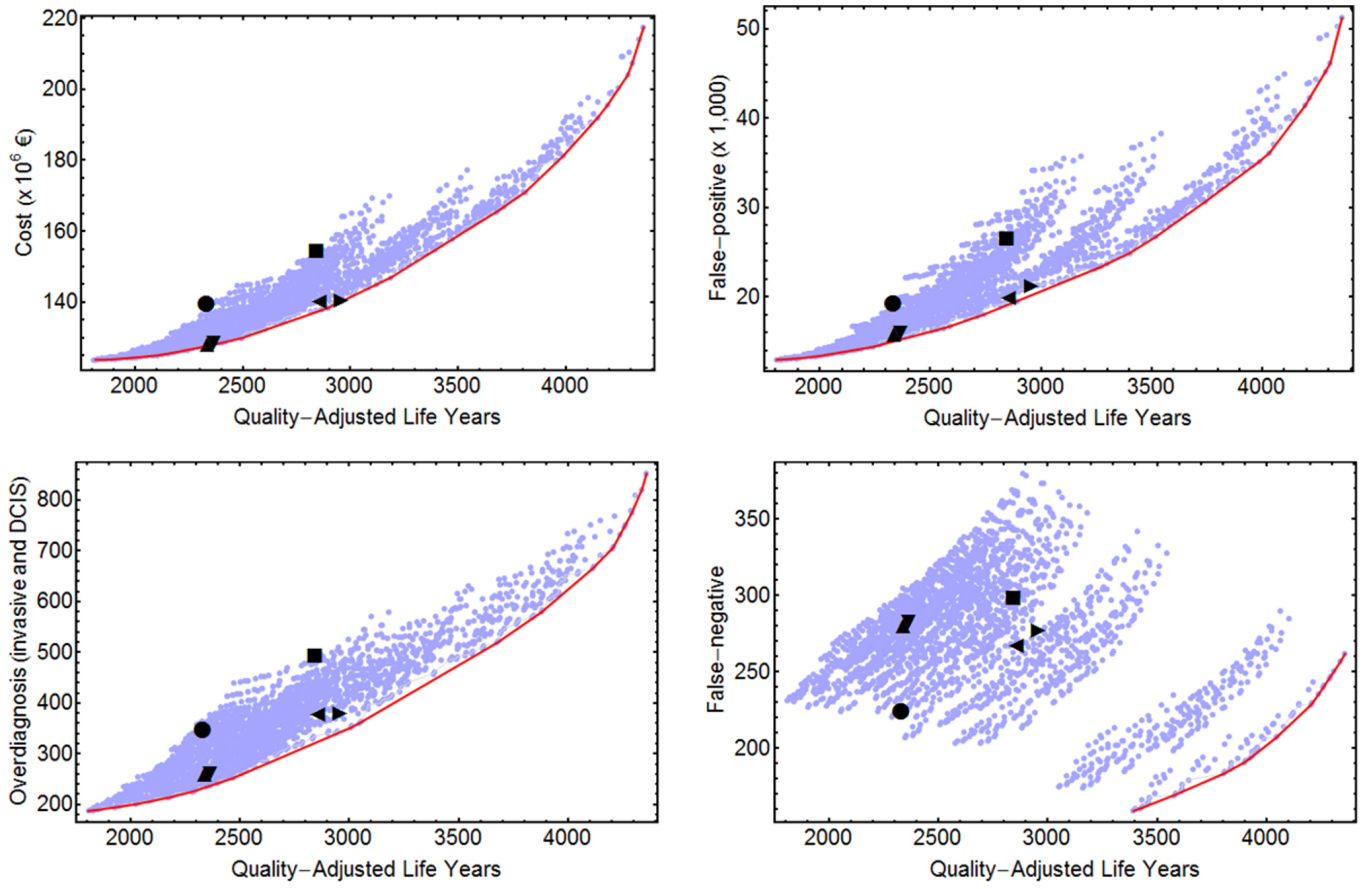
Cost-effectiveness and harm-benefit analyses for 2,625 early detection strategies. Effect measured in quality-adjusted life years. Dots represent specific screening strategies. Results obtained with an annual discount of 3%. •: uniform B5069; ▪: uniform B4574. ▴: risk-based Q5069-Q4574-Q4574-A4574; ▾: risk-based Q5069-Q4574-Q4574-A4074. ◂: risk-based Q5074-Q5074-A4074-A4074; ▸: risk-based Q4574-Q4574-A4574-A4074. Exams periodicities: A = annual, B = biennial, T = triennial, Q = quinquennial. The first two numbers refer to the age at starting the exams and the last two numbers refer to the age at the last exam. In the risk-based strategies, the four strings correspond to the Low, Medium-Low, Medium-High and High risk groups, respectively.

#### Measuring effectiveness with LE

Figure 1 and Table S10 in Appendix S1 present the results of the cost-effectiveness and harm-benefit analyses. Table 1 (A) shows two selected strategies that improve on the B5069 uniform strategy and two that improve on the B4574 uniform strategy. As an example, compared to B5069, the optimal strategy Q5074-Q5074-T5074-A5074 for the L, ML, MH and H risk groups, respectively, has 3.8% higher benefit in terms of LE and achieves reductions of 8.9% in costs, 25.1% in FP and 20.6% in overdiagnosed cases. In absolute numbers, with an annual discount rate of 3% for every 2,000 women screened, the risk-based strategy Q5074-Q5074-T5074-A5074 would extend about the same number of lives (4) as the uniform B5069 strategy but would avoid 1.5 overdiagnosed cases, 97 FP mammograms (six of them ending with a biopsy) and would save 250,000 euros. The only drawback would be one additional FN. If we consider the uniform strategy B4574 as a reference, the risk-based strategy T5074-T5074-A4574-A4574 results in a 5% higher benefit and reductions of 6.8% in costs, 21.9% in FP and 10.1% in overdiagnosed cases.

**Table 1:** Uniform B5069 and B4574 strategies compared with alternative risk-based strategies.

A) Effect measured in lives extended (LE)^1^					
Schedule	LE	Cost (×10^6^€)	False positive^2^	Overdiagnosis^3^	False negative
Uniform B5069	201.9	139.6	19,256.3	347.6	223.9
Risk-based strategies^4^	Percentage of change, compared to fixed B5069				
Q5074-Q5074-Q4574-A4574	0.6	−9.3	−25.1	−25.9	22.7
Q5074-Q5074-T5074-A5074	3.8	−8.9	−25.1	−20.6	20.8
Uniform B4574	264.7	154.5	26,578.5	493.1	298.2
Risk-based strategies^4^	Percentage of change, compared to fixed B4574				
T5069-B5074-A5074-A5074	0.5	−7.7	−23.0	−12.4	−21.6
T5074-T5074-A4574-A4574	5.0	−6.8	−21.9	−10.1	−9.7

B) Effect measured in quality-adjusted life years (QALY)^1^					
Schedule	QALY	Cost (×10^6^€)	False positive^2^	Overdiagnosis^3^	False negative
Uniform B5069	2,333.3	139.6	19,256.3	347.6	223.9
Risk-based strategies^4^	Percentage of change, compared to fixed B5069				
Q5069-Q4574-Q4574-A4574	0.3	−8.3	−18.3	−25.9	24.9
Q5069-Q4574-Q4574-A4074	1.5	−8.0	−17.2	−25.0	26.2
Uniform B4574	2,848.8	154.5	26,578.5	493.1	298.2
Risk-based strategies^4^	Percentage of change, compared to fixed B4574				
Q5074-Q5074-A4074-A4074	0.4	−9.2	−25.3	−23.4	−10.5
Q4574-Q4574-A4574-A4074	4.0	−9.2	−20.4	−23.0	−7.2

1 Data correspond to a cohort of 100,000 women at birth assessed in the age-interval 40–79 years.

All the absolute values have been discounted at an annual rate of 3%.

2 False positive includes both non-invasive and invasive procedures.

3 Overdiagnosis of invasive and DCIS cases.

4 Periodicity and age-interval for Low, Medium-Low, Medium-High and High risk groups, respectively.

Exams periodicities: A = annual, B = biennial, T = triennial, Q = quinquennial. The first two numbers refer to the age at starting the exams and the last two numbers refer to the age at the last exam.

#### Measuring effectiveness with QALYs

Figure 2 and Table S11 in Appendix S1 present the results of the cost-effectiveness and harm-benefit analyses. Table 1 (B) shows that, compared to the B5069 uniform strategy, the risk-based Q5069-Q4574-Q4574-A4074 strategy results in reductions of 8% in costs, 17.2% in FP and 25% in overdiagnosed cases. Similarly, compared to the uniform strategy B4574, the risk-based Q4574-Q4574-A4574-A4074 strategy achieves an increase of 4% in QALYs and reductions of 9.2% in costs, 20.4% in FP and 23% in overdiagnosed cases.

#### False negative results

We have analyzed the incremental ratios of FN results per unit of benefit separately from the other cost-effectiveness or harm-benefit ratios because the pattern of changes in FN results is affected differently by the periodicity of the exams and the age-interval of screening. For instance, moving from uniform B5069 to uniform A5069 reduces the amount of FN by 29%, but moving from uniform B5069 to uniform B4574 increases the amount of FN by 33%. Figures 1 and 2 show that there were no strategies in the lower left part of the incremental FN per incremental benefit analyses and the harm-benefit frontier for FN per LE or per QALY only included annual screening strategies.

The last column of Table 1 shows the percentages of changes in FN results for the selected risk-based strategies with respect to the uniform B5069 and B4574 strategies. Compared to the uniform B5069 strategy, the selected risk-based strategies, which have a similar benefit, have more FN results (20% or more when the measure of benefit is LE, 25% or more when the benefit is measured in QALY). Nevertheless, when considering the uniform B4574 strategy, the selected risk-based strategies not only have less FP results and overdiagnosed cases but also have less FN results. The finding that there are more FN results from risk-based screening compared to uniform B5069 than compared to uniform B4574 is mostly due to the fact that, in general, the selected risk-based strategies screen women until age 74.

#### Summary of optimal strategies

When all the risk-based strategies that are at or near the Pareto frontier are considered and benefit is measured as LE, the risk-based strategies that provide a similar benefit than the B5069 strategy are caracterized by quinquennial for the L and ML, triennial for the MH and tri-, bi- or annual periodicities for the H risk groups. When benefit is measured as QALYs, the risk-based strategies are characterized by quinquennial periodicities for the L, ML and MH and annual for the H risk groups. When the standard of comparison is the uniform strategy B4574, the risk-based strategies that provide similar benefits, either LE or QALY, are characterized by quinquennial for the L, triennial for the ML, and annual periodicities for the MH and the H risk groups.

Figures S4 and S5 in section G of Appendix S1 show how the uniform screening strategies, other than B5069 and B4574, performed in the cost-efectiveness and harm-benefit analyses.

### Validation of the model inputs

When we assumed a scenario without screening, for the age interval 0 to 74 years, we obtained a cumulative incidence of BC equal to 5.8% and a mortality rate from BC equal to 1.5%. These values were consistent with the literature [36], [37]. Section G.1, and Tables S12 and S13 in Appendix S1 compare our results for the biennial strategy B4569 with the results obtained in the INCA study. The detection rates obtained with our model are slightly higher than the INCA rates for both types of detection (screening or interval), except for the 44–49 age group. The overall program sensitivity was very similar (68.1% in the INCA study versus 68.4% in our model). The stage distributions of the models, either screen-detected or interval, were more favorable than the cases in the INCA study. Table 2 shows the distribution of the interval cases in true interval and FN, by time since last mammogram. The timing of overall interval cases and true interval cases was similar in the INCA study and our model. We observed differences in the distribution of FN results at first and second year after the exams. While in the INCA study there was a higher proportion of FN in the second year, our model had a higher proportion of FN in the first year of the interval. Table 3 compares the overall benefit and harm results for the uniform strategies B5069 and B4574 with published reviews [33]–[35]. We observe similarities between the Lancet review for mortality reduction and with the Cochrane and Euroscreen reviews for overdiagnosis. The ratios of overdiagnosed per LE for the B5069 and B4574 strategies in our study were 1.3 and 1.4, respectively, in the lower range of the reviews.

**Table 2:** Distribution of the interval cases by time since last mammogram.

Time since last mammogram (months)	Interval cancer		True interval and minimal signs		False negative and occult tumors	
	N	%	N	%	N	%
The INCA study^1^						
0–11	420	32.4	142	26.2	117	38.7
12–23	876	67.6	399	73.8	185	61.3
Probabilistic model, biennial screening						
0–11	529	35.3	287	26.8	242	56.5
12–23	971	64.7	785	73.2	186	43.5

1 The total number of interval cases in the INCA study is higher than the sum of true interval and FN, occult and minimal signs, because 60.3% of all the interval cases were reviewed.

**Table 3:** Comparison with published reviews.

	Our study^1^		Independent UK Panel on Breast Cancer Screening review [34]	Cochrane systematic review^2^ [33]	Euroscreen review^3^ [35]
	B5069	B4574			
Mortality reduction (%)	14.4	19.6	20.0	15.0	23.0–30.0
Deaths averted	4.3	5.8	4.3	0.5	7–9
Overdiagnosis	5.5	8.1	12.9	5.0	4
Non invasive FP	265.5	347.8	-	>100	200
Invasive FP	24.9	28.7	-	-	30
Number needed to screen to extend 1 live	233	172	235	2000	111–143

Benefits and harms per 1,000 women screened.

1 time horizon 40–79 years.

2 10 years of follow-up.

3 time horizon 50–79 years.

### Sensitivity analysis

Figures S6 and S7 in Appendix S1 show that if there was a migration of women to higher risk groups, the selected risk-based strategies would achieve even higher benefit than the uniform B5069 and B4574 strategies at similar cost and harm values.

Tables S14 and S15 in Appendix S1 present the results of the sensitivity analysis, when the assumptions on the overdiagnosis rates for invasive BC and DCIS, on the costs of cancer treatment, and on the disutility by FP were changed. Tables S14 and S15 also show the relative changes with respect to the uniform B5069 strategy. In general, the cost-benefit and the harm-benefit analyses were robust to changes in the inputs, but we observed changes in the incremental cost-benefit or harm-benefit ratios. When the overdiagnosis rate of invasive or DCIS tumors increased, the incremental cost- or harm-benefit ratios also increased which means that the cost or the harm for each additional unit of benefit was higher. When treatment costs increased, a reduced number of the strategies located in the left part of the frontier were not optimal anymore. This phenomenon was common to both benefit measures (LE and QALY) and was more marked for a 5-fold than for a 2-fold increase. Finally, when the disutility of FP results increased, the optimal strategies were similar, but the incremental FP per incremental QALY also increased. Section G.2 in Appendix S1 includes further details of the sensitivity analyses.

## Discussion

Our analysis aimed to be a global assessment of the impact that a new paradigm of screening would have on benefit, costs and harms rather than a detailed guideline of how personalized screening should be done.

Using probabilistic models, we have found that risk-based screening strategies are more efficient and have lower harm-benefit ratios than uniform strategies. If, instead of screening biennially all women 50 to 69 years old, we combined quinquennial, triennial and annual exam periodicities for women at L or ML, MH, and H risk, respectively, in the age interval 50 to 74, we would avert the same number of deaths. Similarly, strategies that combine quinquennial exams for women at L or ML risk with annual exams for women at MH or H risk, respectively, in the age interval 45 to 74, result in similar gain in QALYs than the uniform biennial strategy in the age interval 45 to 74. But, the important result is that in both cases the risk-based strategies would result in remarkable reductions of costs, FP results and overdiagnosis.

It is important to notice that a risk-based screening strategy Q5074-Q5074-Q4574-A4574 has similar benefits and less costs and harms than the uniform B5069. This does not mean that Q5074-Q5074-Q4574-A4574 should be recommended, only that the same benefits as B5069 can be achieved more efficiently and safely. In fact, in terms of LE, Q5074-Q5074-T5074-A5074 improves the uniform B5069 and has similar costs and harms to Q5074-Q5074-Q4574-A4574. The cost-effectiveness and harm-benefit analyses show the trade-offs when moving along the Pareto frontier. Drawing horizontal lines at the level of uniform strategies, one can estimate the improvement in benefit for a specific cost or harm. Drawing vertical lines allows estimation of the reduction in costs or harms for a specific benefit.

Some recent works have proposed personalized recommendations for BC screening based on cost-effectiveness or cost-utility analyses [8], [9] or in decision models that compare harm and benefits [38]. Schousboe *et al.* [8] established cost-effectivenes thresholds of $100,000 or $50,000 per QALY gained and compared different periodicities for the screening exams in 10-year age groups, BI-RADS breast density categories and the presence/absence of personal history of biopsy and family history of breast cancer. They recommended that women aged 50 to 79 years who have low breast density and no other breast cancer risk factors may consider having mammography less frequently than every 2 years, which is consistent with our results. But, they recommended biennial screening for women aged 50 to 79 with breast densities of 3 or 4, independently of the presence of the other two risk factors. In our study, women with breast density 3 or 4 belong to ML, MH, and H risk groups, depending on having 0, 1 or 2 additional risk factors, respectively, and therefore the optimal strategy would have recommended different periodicities and age intervals for these three risk groups. In addition, Schousboe *et al.* concluded that annual mammography was not cost-effective for any group, regardless of age or breast density. These recommendations do not agree with our results, probably due to differences in the studies' objectives and the methodological approaches used.

van Ravesteyn *et al.* used different models - one of them was the LZ model that we used in the present study - to assess the false-positive mammography findings per death averted and per years of life gained in women aged 40 to 49 years [9]. In all models, screening women with increased risk for breast cancer lead to more breast cancer deaths averted with approximately the same number of false-positive results.

Ayer *et al.* [38], using a Markov decision process that considers personal risk characteristics and the personal history of screening, showed that personalized screening strategies outperform the existing guidelines with respect to the total expected quality-adjusted life years, while significantly decreasing the number of mammograms and false-positives. They concluded that screening is less beneficial for most women over age 74 and, as we found, provides significant QALY gains, for the high-risk women in the age group 40–49.

### Limitations and other considerations

We have used a very detailed model that allowed us to thoroughly assess the cost-effectiveness and harm-benefit of 2,625 different screening scenarios, either risk-based or not. However, our study has several limitations.

First, our model relies on data and assumptions that may be not correct. When available, we have used Catalan or Spanish data from population based registries or BC screening programs. If the input data was not available at the region or country level, we used data that the Cancer Intervention and Surveillance Modeling Network (CISNET) had prepared for BC mortality modeling research groups in the USA, like the distribution of disease stages at diagnosis [12], or from the Breast Cancer Surveillance Consortium, like the distribution of risk factors in the population or the relative risks of the considered risk factors [8], [20], [22]. Finally, there were some inputs that had been obtained from published randomized clinical trials and observational studies [12], [23]. This variety of data sources and modeling assumptions makes it necessary to carefully analyze the model outputs. To validate our model, on one hand, we have performed sensitivity analyses either in this study or in previous publications that show that the model and results are robust to the model assumptions [10], [16]. On the other hand, we have reviewed the literature to check whether our results were consistent, at least for the screening strategies that have been included in reviews - mostly biennial strategies in the 50–69 year age interval. The three examined reviews, the Cochrane systematic review [33], the Independent UK Panel on Breast Cancer Screening review [34], and the Euroscreen comprehensive review of European screening programs [35] provide a wide range of values for the benefits and harms of screening. Our results have similarities and differences with the three reviews. We obtained a value close to the Lancet review for number of deaths averted per 1,000 women. Our ratios of overdiagnosed cases per death averted were in the low range of values obtained in the mentioned reviews, 0.5, 3 and 10 overdiagnosed cases per death averted in the Euroscreen, the UK Panel and the Cochrane reviews, respectively. Our estimates of false-positive mammography results were higher than in the reviews, nevertheless for invasive false-positives we were close to the Euroscreen result. Finally, when we compared our results for the uniform screening strategies B4569 or B5069 with the INCA study or other studies of interval cancer [39]–[41], we found a high consistency in most of the results relative to the number of cancer cases detected per mammography, sensitivity of the program, distribution of screen-detected and interval cases, and distribution of true interval and false-negative cases.

Second, we have assumed that BC risk influenced only the incidence of the disease and not the distribution of stages at diagnosis, the sensitivity and specificity of mammography, the sojourn time in the preclinical state or the mortality from other causes. It could happen that tumors for women at MH or H risk groups had a less favorable stage distribution at diagnosis and the benefit of screening for these groups was lower than estimated. Also, it is known that mammography performance is associated with the considered risk factors [42], [43].

Third, we have assumed that there are no changes in the risk factors after the age at which screening exams start. We considered that the proportion of women in the risk groups remained constant over time and it was the overall sample estimate for the BCSC data. This assumption may not be correct, because as women get older breast density tends to decrease and personal history of biopsy and family history of breast cancer have more chances to be present. We think that our results are robust to changes in the risk group weights over time, as the sensitivity analysis has shown to be the case for changes in the risk group distributions. However, when considering personalized screening, BC risk should be updated when new information on risk factors or their trends is available.

Forth, our model used age-specific sensitivities of the screening exam that correspond to a more prevalent use of film mammography than digital mammography. We did not assess the impact of changing the mammography performance in this study. van Ravesteyn *et al.* [9] found that there was greater harm relative to benefit from digital than from film mammography in women aged 40–49 years, an age group were it seems that digital mammography has higher sensitivity, detects more cases of DCIS and results in more FP results [44], [45].

Fifth, our probabilistic model assumes that screening results in a stage-shift at BC diagnosis, but does not consider DCIS as one of the BC stages. Therefore, the fraction of DCIS tumors that would have progressed and been diagnosed as invasive in the absence of screening, are re-distributed under screening in more favorable stages at diagnosis, but not as DCIS. This may have produced an underestimation of the benefit of the screening strategies, both uniform or risk-based. If bias had affected uniform and risk-based strategies similarly, the cost-effectiveness and harm-benefit analyses would remain valid.

We agree with Mandelblatt [46] and Ayer [38] on that risk-based approaches show promise, but there are important issues that need further research. One issue is the need to know more about the underlying relationships between risk factors and the biology of breast cancer and, the other issue, is to overcome the practical issues of implementing appropriate screening strategies based on personalized risk. The PROCAS study in the UK [47], the KARMA project in Sweden [48]–[50], and the PROSPR network in the USA [51] are examples of advancing towards a tailored screening through improving BC risk prediction. Creating new strategies for communicating individual estimates of benefit and risk of alternative screening methods, to better inform patients and health care providers, is a challenge for researchers.

In conclusion, risk-based screening strategies seem to be more efficient and have better harm-benefit ratios than the standard uniform strategies. We have proposed a reduced number of risk-based screening strategies that combine quinquennial or triennial exams for women in low or moderate-low risk groups and annual exams for women in the moderate-high or high risk groups, for the consideration of researchers, decision makers and policy planners. Now, it is necessary to develop accurate measures of individual risk of BC and to work on how to organise risk-based screening programs.

## Supporting Information

Appendix S1
**Supporting information on methods used and results obtained, containing Tables S1 to S15 and Figures S1 to S7.** Table S1, Distribution of stages at diagnosis of BC. Table S2, Relative risk of breast cancer based on age and breast density. Table S3, Prevalences of risk factors by age group for each category of breast density. Table S4, Characteristics of the 2,625 screening strategies analized. Table S5, The utilities for the general population and for women diagnosed with BC, either DCIS or invasive. Table S6, Model for false positives of non-invasive tests. Table S7, Model for false positives of invasive tests. Table S8, Distribution of stages at diagnosis of BC for screen-detected cases. Different overdiagnosis rates. Table S9, Linear regression model with dependent variable being the DCIS rate per 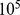 women-year. Table S10, Cost-effectiveness and harm-benefit analysis. Lives extended. Table S11, Cost-effectiveness and harm-benefit analysis. Quality-adjusted life years (QALY). Table S12, Number of mammograms and detection rates for screen-detected and interval cases and program sensitivity by age groups. Invasive cancer (DCIS not included). Table S13, Distribution of stages at diagnosis of BC. Table S14, Sensitivity analysis. Changes in lives extended. Table S15, Sensitivity analysis. Changes in QALY. Figure S1, Incidence curves for twelve risk profiles grouped by risk level: (A) Low Risk, (B) Medium-Low Risk, (C) Medium-High Risk, and (D) High Risk. Graphic (E) shows the smoothed incidence rates for each risk group. Figure S2, Observed and smoothed DCIS rates over time in Catalonia (1983–2008). Figure S3, Index of mammography use (IMU) and smoothed DCIS rates over time in Catalonia (1983–2008). Figure S4, Cost-effectiveness and harm-benefit analyses for 2,625 early detection strategies, with uniform strategies marked. Effect measured in lives extended. Figure S5, Cost-effectiveness and harm-benefit analyses for 2,625 early detection strategies, with uniform strategies marked. Effect measured in QALY. Figure S6, Sensitivity analysis of a change in the risk groups distribution. Cost-effectiveness and harm-benefit analyses for 2,625 early detection strategies. Effect measured in lives extended. Figure S7, Sensitivity analysis of a change in the risk groups distribution. Cost-effectiveness and harm-benefit analyses for 2,625 early detection strategies. Effect measured in QALY.(PDF)

## References

[1] Feuer E, Plevritis SK, Berry DA, Cronin KA, editors. et al The impact of mammography and adjuvant therapy on US breast cancer mortality (1975–2000): collective results from the Cancer Intervention and Surveillance modeling network. J Natl Cancer Inst Monogr 36: 1–126.

[2] Roman R, Sala M, Salas D, Ascunce N, Zubizarreta R, et al (2011) Effect of protocol-related variables and women's characteristics on the cumulative false-positive risk in breast cancer screening. Ann Oncol 23: 104–111. 21430183 10.1093/annonc/mdr032 PMC3276323

[3] Kalager M, Adami HO, Bretthauer M, Tamimi RM (2012) Overdiagnosis of invasive breast cancer due to mammography screening: results from the Norwegian screening program. Ann Intern Med 156: 491–499. 22473436 10.7326/0003-4819-156-7-201204030-00005

[4] Jorgensen KJ, Gotzsche PC (2009) Overdiagnosis in publicly organised mammography screening programmes: systematic review of incidence trends. BMJ 339: b2587. 19589821 10.1136/bmj.b2587 PMC2714679

[5] Mandelblatt JS, Cronin KA, Bailey S, Berry DA, de Koning HJ, et al (2009) Effects of mammography screening under different screening schedules: model estimates of potential benefits and harms. Ann Intern Med 151: 738–747. 19920274 10.1059/0003-4819-151-10-200911170-00010 PMC3515682

[6] Perry N, Broeders M, de Wolf C, Törnberg S, Holland R, et al (2008) European guidelines for quality assurance in breast cancer screening and diagnosis. Fourth edition-summary document. Ann Oncol 19: 614–22. 18024988 10.1093/annonc/mdm481

[7] Joy J, Penhoet E, Petitti D, editors, Institute of Medicine and National Research Council Committee on new approaches to early detection and diagnosis of breast cancer (2005) Saving women's lives: strategies for improving breast cancer detection and diagnosis. The National Academies Press, Washington D.C. Available: http://www.ncbi.nlm.nih.gov/books/NBK22315/. Accessed 2013 Dec 21.

[8] Schousboe JT, Kerlikowske K, Loh A, Cummings SR (2011) Personalizing mammography by breast density and other risk factors for breast cancer: analysis of health benefits and cost-effectiveness. Ann Intern Med 155: 10–20. 21727289 10.7326/0003-4819-155-1-201107050-00003 PMC3759993

[9] van Ravesteyn NT, Miglioretti DL, Stout NK, Lee SJ, Schechter CB, et al (2012) Tipping the balance of benefits and harms to favor screening mammography starting at age 40 years: a comparative modeling study of risk. Ann Intern Med 156: 609–617. 22547470 10.1059/0003-4819-156-9-201205010-00002 PMC3520058

[10] Carles M, Vilaprinyo E, Cots F, Gregori A, Pla R, et al (2011) Cost-effectiveness of early detection of breast cancer in Catalonia (Spain). BMC Cancer 11: 192. 21605383 10.1186/1471-2407-11-192 PMC3125279

[11] Lee S, Zelen M (1998) Scheduling periodic examinations for the early detection of disease: applications to breast cancer. J Am Stat Assoc 93: 1271–1281.

[12] Lee S, Zelen M (2006) A stochastic model for predicting the mortality of breast cancer. J Natl Cancer Inst Monogr 36: 79–86. 17032897 10.1093/jncimonographs/lgj011

[13] Lee SJ, Zelen M (2008) Mortality modeling of early detection programs. Biometrics 64: 386–395. 17725809 10.1111/j.1541-0420.2007.00893.x

[14] Vilaprinyo E, Gispert R, Martinez-Alonso M, Carles M, Pla R, et al (2008) Competing risks to breast cancer mortality in Catalonia. BMC Cancer 8: 331. 19014473 10.1186/1471-2407-8-331 PMC2636833

[15] Vilaprinyo E, Rue M, Marcos-Gragera R, Martinez-Alonso M (2009) Estimation of age- and stage- specific Catalan breast cancer survival functions using US and Catalan survival data. BMC Cancer 9: 98. 19331670 10.1186/1471-2407-9-98 PMC2679763

[16] Rue M, Vilaprinyo E, Lee S, Martinez-Alonso M, Carles M, et al (2009) Effectiveness of early detection on breast cancer mortality reduction in Catalonia (Spain). BMC Cancer 9: 326. 19754959 10.1186/1471-2407-9-326 PMC2758899

[17] Martinez-Alonso M, Vilaprinyo E, Marcos-Gragera R, Rue M (2010) Breast cancer incidence and overdiagnosis in Catalonia (Spain). Breast Cancer Res 12: R58. 20682042 10.1186/bcr2620 PMC2949650

[18] Zelen M, Feinleib M (1969) On the theory of screening for chronic diseases. Biometrika 56: 601–614.

[19] Day NE, Walter SD (1984) Simplified models of screening for chronic disease: estimation procedures from mass screening programmes. Biometrics 40: 1–14. 6733223

[20] Tice JA, Cummings SR, Smith-Bindman R, Ichikawa L, Barlow WE, et al (2008) Using clinical factors and mammographic breast density to estimate breast cancer risk: development and validation of a new predictive model. Ann Intern Med 148: 337–347. 18316752 10.7326/0003-4819-148-5-200803040-00004 PMC2674327

[21] American College of Radiology (2003) The American College of Radiology Breast Imaging Reporting and Data System (BI-RADS). American College of Radiology, Reston (VA).

[22] Barlow WE, White E, Ballard-Barbash R, Vacek PM, Titus-Ernstoff L, et al (2006) Prospective breast cancer risk prediction model for women undergoing screening mammography. J Natl Cancer Inst 98: 1204–1214. 16954473 10.1093/jnci/djj331

[23] Lidgren M, Wilking N, Jonsson B, Rehnberg C (2007) Health related quality of life in different states of breast cancer. Qual Life Res 16: 1073–1081. 17468943 10.1007/s11136-007-9202-8

[24] Biesheuvel C, Barratt A, Howard K, Houssami N, Irwig L (2007) Effects of study methods and biases on estimates of invasive breast cancer overdetection with mammography screening: a systematic review. Lancet Oncol 8: 1129–1138. 18054882 10.1016/S1470-2045(07)70380-7

[25] de Koning HJ, Draisma G, Fracheboud J, de Bruijn A (2006) Overdiagnosis and overtreatment of breast cancer: microsimulation modelling estimates based on observed screen and clinical data. Breast Cancer Res 8: 202. 16524452 10.1186/bcr1369 PMC1413979

[26] Morrell S, Barratt A, Irwig L, Howard K, Biesheuvel C, et al (2009) Estimates of overdiagnosis of invasive breast cancer associated with screening mammography. Cancer Causes Control 21: 275–282. 19894130 10.1007/s10552-009-9459-z

[27] Zackrisson S, Andersson I, Janzon L, Manjer J, Garne JP (2006) Rate of over-diagnosis of breast cancer 15 years after end of Malmo mammographic screening trial: follow-up study. BMJ 332: 689–692. 16517548 10.1136/bmj.38764.572569.7C PMC1410836

[28] Zahl PH, Maehlen J, Welch HG (2008) The natural history of invasive breast cancers detected by screening mammography. Arch Intern Med 168: 2311–6. 19029493 10.1001/archinte.168.21.2311

[29] Rue M, Carles M, Vilaprinyo E, Martinez-Alonso M, Espinas JA, et al (2008) Dissemination of periodic mammography and patterns of use, by birth cohort, in Catalonia (Spain). BMC Cancer 8: 336. 19014679 10.1186/1471-2407-8-336 PMC2613154

[30] Perez MJ, Gregori A, Carles M, Gispert R, Martinez-Alonso M, et al (2010) The evolution of breast cancer mortality and the dissemination of mammography in Catalonia: an analysis by health region. Rev Esp Salud Publica 84: 691–703. 21327306 10.1590/s1135-57272010000600002

[31] Erbas B, Amos A, Fletcher A, Kavanagh AM, Gertig DM (2004) Incidence of invasive breast cancer and ductal carcinoma in situ in a screening program by age: should older women continue screening? Cancer Epidemiol Biomarkers Prev 13: 1569–1573. 15466971

[32] Lopez Bastida J, Oliva J, Antonanzas F, Garcia-Altes A, Gisbert R, et al (2010) A proposed guideline for economic evaluation of health technologies. Gac Sanit 24: 154–170. 19959258 10.1016/j.gaceta.2009.07.011

[33] Gotzsche PC, Jorgensen K (2013) Screening for breast cancer with mammography. Cochrane Database Syst Rev 6: CD001877. 10.1002/14651858.CD001877.pub5 PMC6464778 23737396

[34] Independent UK Panel on Breast Cancer Screening (2012) The benefits and harms of breast cancer screening: an independent review. Lancet 380: 1778–1786. 23117178 10.1016/S0140-6736(12)61611-0

[35] Paci E (2012) EuroscreenWorking Group (2012) Summary of the evidence of breast cancer service screening outcomes in Europe and first estimate of the benefit and harm balance sheet. J Med Screen 19 Suppl 1: 5–13. 22972806 10.1258/jms.2012.012077

[36] Ferlay J, Shin HR, Bray F, Forman D, Mathers C, et al (2010) Estimates of worldwide burden of cancer in 2008: GLOBOCAN 2008. Int J Cancer 127: 2893–2917. 21351269 10.1002/ijc.25516

[37] Izquierdo A, Gispert R, Saladie F, Espinas JA (2008) Analysis of cancer incidence, survival and mortality according to the main tumoral localizations, 1985–2019: Breast cancer. Med Clin (Barc) 131 Suppl 1: 50–52. 19080815 10.1016/s0025-7753(08)76433-9

[38] Ayer T, Alagoz O, Stout N (2012) A POMDP Approach to Personalize Mammography Screening Decisions. Operations Research 60: 1019–1034.

[39] McCann J, Britton PD, Warren RM, Hunnam G (2001) Radiological peer review of interval cancers in the East Anglian breast screening programme: what are we missing? East Anglian Breast Screening Programme. J Med Screen 8: 77–85. 11480448 10.1136/jms.8.2.77

[40] Domingo L, Sala M, Servitja S, Corominas JM, Ferrer F, et al (2010) Phenotypic characterization and risk factors for interval breast cancers in a population-based breast cancer screening program in Barcelona, Spain. Cancer Causes Control 21: 1155–1164. 20349271 10.1007/s10552-010-9541-6

[41] Kirsh VA, Chiarelli AM, Edwards SA, O'Malley FP, Shumak RS, et al (2011) Tumor characteristics associated with mammographic detection of breast cancer in the ontario breast screening program. J Natl Cancer Inst 103: 942–950. 21540443 10.1093/jnci/djr138

[42] Kerlikowske K, Carney PA, Geller B, Mandelson MT, Taplin SH, et al (2000) Performance of screening mammography among women with and without a first-degree relative with breast cancer. Ann Intern Med 133: 855–863. 11103055 10.7326/0003-4819-133-11-200012050-00009

[43] Carney PA, Miglioretti DL, Yankaskas BC, Kerlikowske K, Rosenberg R, et al (2003) Individual and combined effects of age, breast density, and hormone replacement therapy use on the accuracy of screening mammography. Ann Intern Med 138: 168–175. 12558355 10.7326/0003-4819-138-3-200302040-00008

[44] Pisano ED, Gatsonis C, Hendrick E, Yaffe M, Baum JK, et al (2005) Diagnostic performance of digital versus film mammography for breast-cancer screening. N Engl J Med 353: 1773–1783. 16169887 10.1056/NEJMoa052911

[45] Karssemeijer N, Bluekens AM, Beijerinck D, Deurenberg JJ, Beekman M, et al (2009) Breast cancer screening results 5 years after introduction of digital mammography in a population-based screening program. Radiology 253: 353–358. 19703851 10.1148/radiol.2532090225

[46] Mandelblatt JS, Stout N, Trentham-Dietz A (2011) To screen or not to screen women in their 40s for breast cancer: is personalized risk-based screening the answer? Ann Intern Med 155: 58–60. 21727294 10.7326/0003-4819-155-1-201107050-00008

[47] National Health Service (no date) The PROCAS Study website. Available: http://www.uhsm.nhs.uk/research/Pages/PROCASstudy.aspx. Accessed 2013 Dec 21.

[48] Karolinska Institutet (no date) Karolinska Mammography Project for Risk Prediction of Breast Cancer website. Available: http://karmastudy.org/. Accessed 2013 Dec 21.

[49] Darabi H, Czene K, Zhao W, Liu J, Hall P, et al (2012) Breast cancer risk prediction and individualised screening based on common genetic variation and breast density measurement. Breast Cancer Res 14: R25. 22314178 10.1186/bcr3110 PMC3496143

[50] Li J, Szekely L, Eriksson L, Heddson B, Sundbom A, et al (2012) High-throughput mammographicdensity measurement: a tool for risk prediction of breast cancer. Breast Cancer Res 14: R114. 22846386 10.1186/bcr3238 PMC3680940

[51] National Cancer Institute (no date) Cancer Control Research website. Available: http://cancercontrol.cancer.gov/grants/abstract.asp?applid=8223497. Accessed 2013 Dec 21.

