# Supplementary material for: Cost-Effectiveness and Harm-Benefit Analyses of Risk-Based Screening Strategies for Breast Cancer
Source: PLoS One. 2014 Feb 3;9(2):e86858. doi: 10.1371/journal.pone.0086858 (PMC3911927; doi:10.1371/journal.pone.0086858)
Supplement: Appendix S1 — Supporting information on methods used and results obtained, containing Tables S1 to S15 and Figures S1 to S7. Table S1, Distribution of stages at diagnosis of BC. Table S2, Relative risk of breast cancer based on age and breast density. Table S3, Prevalences of risk factors by age group for each category of breast density. Table S4, Characteristics of the 2,625 screening strategies analized. Table S5, The utilities for the general population and for women diagnosed with BC, either DCIS or invasive. Table S6, Model for false positives of non-invasive tests. Table S7, Model for false positives of invasive tests. Table S8, Distribution of stages at diagnosis of BC for screen-detected cases. Different overdiagnosis rates. Table S9, Linear regression model with dependent variable being the DCIS rate per women-year. Table S10, Cost-effectiveness and harm-benefit analysis. Lives extended. Table S11, Cost-effectiveness and harm-benefit analysis. Quality-adjusted life years (QALY). Table S12, Number of mammograms and detection rates for screen-detected and interval cases and program sensitivity by age groups. Invasive cancer (DCIS not included). Table S13, Distribution of stages at diagnosis of BC. Table S14, Sensitivity analysis. Changes in lives extended. Table S15, Sensitivity analysis. Changes in QALY. Figure S1, Incidence curves for twelve risk profiles grouped by risk level: (A) Low Risk, (B) Medium-Low Risk, (C) Medium-High Risk, and (D) High Risk. Graphic (E) shows the smoothed incidence rates for each risk group. Figure S2, Observed and smoothed DCIS rates over time in Catalonia (1983–2008). Figure S3, Index of mammography use (IMU) and smoothed DCIS rates over time in Catalonia (1983–2008). Figure S4, Cost-effectiveness and harm-benefit analyses for 2,625 early detection strategies, with uniform strategies marked. Effect measured in lives extended. Figure S5, Cost-effectiveness and harm-benefit analyses for 2,625 early detection strategies, with uniform strategies [file pone.0086858.s001.pdf]

Appendix S1

A The probabilistic model

Lee and Zelen (LZ) developed a probabilistic model that predicts mortality as a function of the early detection modality. The characteristics and assumptions of the LZ model are described in detail elsewhere [1–3]. The assumptions of the LZ model are (1) a four-state progressive disease in which a subject may be in a disease-free state (S_0), preclinical disease state (S_p: capable of being diagnosed by a special exam), clinical state (S_c: diagnosis by symptomatic detection), and a death from breast cancer (BC) state (S_d); (2) age-dependent transitions into the different states; (3) age-dependent examination sensitivity; (4) age-dependent sojourn times in each state; and (5) exam-diagnosed cases have a stage-shift in the direction of more favorable prognosis relative to the distribution of stages in symptomatic detection.

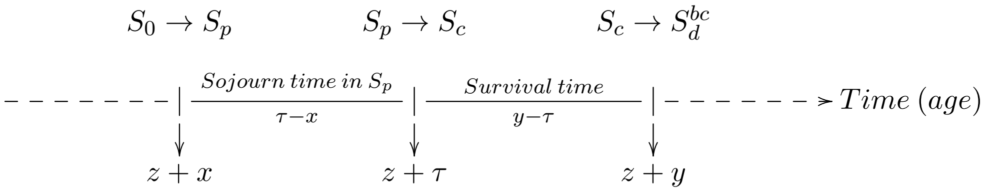

The LZ model considers:

- n screening exams at times $t_0 < t_1 < ... < t_{n-1}$. It is assumed that $t_0 = 0$ and $z = age$ at t_0.
- Three chronological times (see schema):
  - x: time at entering S_p, $z + x$: age when entering S_p. The time x is not observed but can be derived from the incidence function and the distribution of sojourn time in the S_p state. x takes a negative value if the transition to S_p occurs before the age at first exam, z.
  - τ: time at entering S_c, $z + \tau$: age at entering S_c. The time τ can not be observed in cases detected by exam, only in the clinically detected cases. For cases detected by exam, τ can be estimated.
  - y: time at death, $z + y$: age at death. Then $x < \tau < y$

- Sojourn time in S_p: $\tau - x$
- Sojourn time in S_c: $y - \tau$

The LZ basic model calculates the cumulative probability of death for the cohort group exposed to any screening program after T years of follow-up. Similarly, the cumulative probability of death for the cohort group receiving typical health care can be calculated. These probabilities are used to calculate possible reductions in mortality in an early detection program after T years of follow-up.

Survival distributions for exam-diagnosed, interval, and control cases are assumed to be conditional on the stage at diagnosis and treatment, but are not dependent on the mode of diagnosis. The LZ model assumes k stages, $\phi_s(j)$, $\phi_i(j)$ and $\phi_c(j)$ represent the probability of being diagnosed at stage j, $j = 1, ..., k$ for exam-diagnosed, interval and control cases, respectively, and $f_j(t|z+\tau)$ is the probability density function (pdf) of survival time t among subjects who would have been clinically diagnosed at stage j in the absence of screening. Then the survival time pdfs of the exam-diagnosed, interval and control cases are the mixtures: $g_s(t|z+\tau) = \sum_{j=1}^{k} \phi_s(j) f_j(t|z+\tau)$, $g_i(t|z+\tau) = \sum_{j=1}^{k} \phi_i(j) f_j(t|z+\tau)$ and $g_c(t|z+\tau) = \sum_{j=1}^{k} \phi_c(j) f_j(t|z+\tau)$, respectively.

Since screening will appear to increase survival time, the LZ model controls for *lead time* bias by setting the origin of survival time for the screened, interval, and clinical cases at the time of clinical diagnosis. Consequently, there is an implied *guarantee time* for disease-specific survival, that is, the cases diagnosed earlier would have been alive at the time the disease would have been clinically diagnosed. This guarantee time, also called *lead time*, is a random variable and is incorporated into the equations of the model. Explicitly, the lead time is $\tau - t_r$ where τ is the time at which the individual enters the clinical state and t_r is the time at which the r detection exam, when the disease will be diagnosed, is given.

A.1 Survival functions

Since the BC survival functions in presence of screening are affected by the lead time and length biases, we used the Catalan BC survival functions by age and stage for the period 1980-89 [4], which correspond to the pre-screening era in Spain. To introduce the benefit of BC treatments

during the 1990s and the 2000s, we multiplied the 1980-89 BC hazard rates by stage-specific hazard ratios (HR) found in the literature. For local and regional BC, the hazard ratios of adjuvant treatment - either multi-chemotherapy or hormonal treatment - were adapted from Mariotto *et al.* [5]. We assumed HR=0.85 for localized BC (AJCC stages I or II-) and HR=0.8 for regional BC (stages II+ and III). For metastatic BC we assumed HR=0.72 [6].

A.2 Distribution of stages at diagnosis

Table S1 presents the distribution of stages at diagnosis for screen-detected, interval and background cancer cases, according to periodicity of exams. The stage distribution for cases without screening was obtained from the Surveillance Epidemiology and End Results (SEER) program of the National Cancer Institute and the stage distribution for the screen-detected and interval cases from the Breast Cancer Surveillance Consortium (BCSC), by Lee and Zelen [2]. For screen-detected cases and interval cases Lee and Zelen distinguished between annual, biennial and irregular screening.

The stage distributions of screen-detected cases in Table S1 were modified to deal with assumptions of overdiagnosis in the main analysis as well as in the sensitivity analysis. (See section E and Table S8 of this Appendix S1).

B Invasive BC incidence by risk group

B.1 Overall incidence model for invasive BC

To estimate the age-specific incidence rates for invasive BC we used the model published elsewhere by Martinez-Alonso *et al.* [7]. This model estimates the expected number of incident cases, $E(I)$, using the equation:

$$E(I) = \exp(-6.0626 - 38.4818\ Age_1 + 0.0005\ Age_2 + 0.6250\ PM50_1 + 0.0120\ YB_1 + \text{offset})$$

where $Age_1 = (age/10)^{-2} - 0.0331$, $Age_2 = (age/10)^3 - 166.375$, $PM50_1 = PM50^{0.5} - 0.4342$ (with *PM50* being the proportion of women who were having periodic mammograms for early detection at age 50), $YB_1 = \text{year-of-birth} - 1937.5$ and offset $= \log(\text{exposed}) - 0.15\,CFR$, where *CFR* indicates the complete fertility rate of the cohort. To obtain the background incidence we assumed *PM50*=0.

B.2 BC incidence in risk groups

BC risk groups were defined according to the following variables: breast density (measured using the BI-RADS categories 1 to 4 [8]), family history of BC in first degree relatives (yes/no) and personal history of breast biopsy (yes/no). Relative risks of invasive breast cancer based on breast density, family history of BC, and breast biopsy were taken from Tice *et al.* [9] and Schousboe *et al.* [10].

To obtain the age-specific BC incidence by risk group we took the following steps:

1. We estimated the age-specific BC incidence for each breast density category based on the reported relative risks from Schousboe *et al.* [10]. Table S2 shows, by age groups, the relative risks for each breast density level compared to all women in the corresponding age group.

2. Based on data from Tice *et al.* [9], we approximated the relative risks of invasive breast cancer to 1.5 or 0.9, for the presence or absence of a family history of BC in a first degree relative, respectively. The same relative risks, 1.5 or 0.9, were assumed for a positive or negative history of prior breast biopsy.

3. With the previous considerations, twelve detailed age-specific incidence curves were obtained. We grouped them in four aggregated age-specific incidence curves taking into account the proximity of the detailed curves. The four incidence groups represented: (i) category 1 breast density with at most one risk factor; and category 2 breast density with no risk factors (Low Risk); (ii) category 1 breast density with two risk factors; and category 2 breast density with one risk factor; and category 3, 4 breast density with no risk factors (Medium-Low Risk); (iii) category 2 breast density with two risk factors; and category 3, 4 breast density with one risk factor (Medium-High Risk); and category 3, 4 breast density with two risk factors (High Risk).

4. The incidence rates of the four aggregated risk groups were estimated as weighted sums of the detailed incidence curves. The weights were based on the prevalences of each combination of risk factors obtained from the Risk Estimation Dataset of the Breast Cancer Surveillance Consortium (BCSC) [11]. Figure S1 shows the detailed and aggregated incidence curves.

Table S3 shows the prevalences of risk factors by age group for each level of breast density.

C Quality Adjusted Life Years (QALY)

The QALYs for the different breast cancer disease states were obtained using the health related quality of life (HRQoL) measures from Lidgren *et al.* [12]. As Schousboe *et al.* [10], we used the HRQoL measures obtained from the EuroQoL EQ-5D self classifier in the Lidgren *et al.*'s study, which provided HRQoL measures for the first year after primary BC (EQ-5D=0.696); the first year after recurrence (EQ-5D=0.779); the second and following years after primary BC or recurrence (EQ-5D=0.779); and the metastatic BC state (EQ-5D=0.685).

We also followed Schousboe *et al.* [10] to obtain the QALY values for the first year after BC diagnosis: 0.744 for ductal carcinoma in situ (DCIS), 0.696 for localized BC and 0.620 for regional or distant BC. For women that did not die of BC, beyond the first year of diagnosis and during four years, we assumed no loss in quality of life for women with DCIS and a QALY value of 0.779 for the invasive cancers, either local, regional or distant at diagnosis. For women that died of breast cancer, we considered that the last four years of their lives were spent in a distant stage with a QALY value of 0.685, independently of the stage at diagnosis.

The HRQoL data of the Lidgren *et al.*'s study were not reported by age. To obtain the QALY values by age group and BC state we replicated the work of Schousboe *et al.* [10]. The estimated average QALY value of Swedish women at the mean age of the Lidgren's population was 0.823. The proportion of expected quality of life, for any given age for all of the BC states, compared to perfect health (QALY = 1.0) was obtained dividing the previously stated values for each BC state by 0.823. The final QALY value, for each BC state and for any given age group, was obtained multiplying these proportions by the QALY value for that age group in the general population. The QALY values that we used are shown in table S5.

For false-positive mammograms we assumed an average annualized loss of quality of life of 0.013. Schousboe *et al.* [10] reported that they used this value in their sensitivity analysis but assumed no loss in quality of life in their main analysis. To obtain the 0.013 value they assumed that 50% of women with a false-positive would have anxiety sufficient to increase the mood subscale of the EuroQol instrument from 0 to 1, lasting a total of 2 months. According to the U.S. EQ-5D tariffs,

such a change for an entire year represents a decrease in the QALY value of 0.156, which divided by 12 (1/2 women $\times$ 1/6 years) is 0.013. In the sensitivity analysis we assessed the impact of changing the disutility by false-positive to 0, and to 0.026.

D Model that estimates the false-positive rates for invasive and non-invasive tests

The Cumulative False Positive Research study (RAFP) provided the false-positive (FP) rates for invasive and non-invasive tests for women starting biennial exams at ages 44-45 to 68-69. The maximum number of screening rounds was six (non-published data).

We used the FP rate as the dependent variable and included the screening round and age as independent variables, in a log linear model. Tables S6 and S7 contain the parameter estimates. The model allowed us to estimate the FP rate for annual screening. We assumed that after the 6th exam the FP rate was constant. Tables S6 and S7 show the estimated coefficients, confidence intervals and p-values.

E Distribution of stages for screen-detected cases under the assumption of overdiagnosis of invasive BC

To re-calculate the stages' distribution of screen-detected cases, first we subtracted the overdiagnosis rate (e.g 15% for the main analysis) from the proportion of stage I of BC. Then, we divided the proportions of each stage by 1-overdiagnosis rate and obtained the new distribution of stages' distribution. Table S8 presents the stages' distributions for screen detected cases used in the main analysis as well in the sensitivity analyses.

As an example, the stages' distribution for annual screen detected cases under the assumption of 15% overdetection rate, for the age-group 40-49 years, has been obtained as follows:

- From Table S1 the proportion in stage I is 0.62. If we subtract 0.15, we obtain a proportion of 0.62-0.15=0.47 women in stage I.
- We subtract 0.15 from 1 and obtain a total of 0.85 women diagnosed with invasive cancer.
- We divide the new proportion for stage I and the old proportions for stages II to IV by 0.85. We obtain 0.47/0.85=0.5529 women in stage I, 0.1131/0.85=0.1331 in stage II-,

0.2141/0.85=0.2519 in stage II+, 0.0436/0.85=0.0513 in stage III and 0.0092/0.85= 0.0108 in stage IV, as indicated in Table S8.

F DCIS attributable to screening

Figure S2 shows the age-specific DCIS rates over time, using the data from the Cancer Registries of the Girona and Tarragona provinces in Catalonia. We used natural splines to obtain a smooth trajectory of the DCIS rates in the age groups 40-49, 50-69 and 70 or more years.

An increase in the DCIS rates, starting at the beginning of the 1990s was observed in the three age groups. In the 40-49 and 50-69 year age groups, we observed a plateau near the year 2000 followed by a new increase afterwards. The DCIS in the older age group seemed to have stabilized during the 2000s.

Our purpose was to quantify the increase in the DCIS rate in relation to the dissemination of mammography for early detection. First, we estimated an index of mammography use (IMU). The data was obtained, for the Girona and Tarragona provinces, from three health surveys performed in the years 1994, 2002 and 2006. Data from these surveys was already analyzed to study the dissemination of periodic mammography in Catalonia [13, 14]. For each health survey year, the IMU was obtained using the following formula:

$$IMU = \sum_{i \in \{1,2,4\}} f_i \times 1/i$$

where f_i is the fraction of women in the population that received early detection mammograms with a periodicity of i years, with $i = 1, 2, 4$ if the interval between mammograms was 1, 2 or 3 or more years, respectively. The IMU measures the intensity of mammography use per woman-year. If no women in the population used periodic mammography, IMU=0. If every woman in the population received an annual mammography, IMU=1 and if half of the women in the population receive a biennial mammograph, IMU= 0.25.

Then, we used a linear regression model to assess the impact of mammography use in the DCIS rate. Age-group was included in the model as a categorical variable. For this model, we used the DCIS rates corresponding to a baseline period (years 1983-1987) and three periods of three years centered around the health survey years, 1993-1995, 2001-2003 and 2005-2007. For the baseline

period, when there was very little use of mammography, it was assumed that the IMU=0.

Figure S3 presents the smoothed DCIS rates and the IMU values. It can be observed that the pattern of increase of the DCIS rate is related to the pattern of growth of the IMU, mainly for the 50-69 age group, which is the target population for the public screening program in Catalonia.

Table S9 presents the results of the linear regression model. The IMU is associated with the DCIS rate with a beta coefficient equal to 31.13, indicating an increase of approximately 31.13 DCIS cases per 100,000 women-year if the IMU changes from 0 (no screening) to 1 (all women receive a mammography). The R^2 coefficient of determination is 0.87, denoting that the model explains a high percentage of the total variability in the DCIS rate.

G Results

Tables S10 and S11 show the Pareto efficiency frontiers of cost-effectiveness and harm-benefit analyses in terms of lives extended (LE) and quality-adjusted life years gained (QALY), respectively.

Figures S4 and S5 contain the same data as Figures 1 and 2 with all the studied uniform strategies labeled.

G.1 Validation of the model inputs

Table S12 compares the screen-detected and interval cancer rates per 1,000 mammograms in the INCA study and the fixed B4569 strategy of the mathematical model. The model detection rates are slightly higher than the INCA rates for both types of detection (screening or interval), except for the 44-49 age group. The age-specific interval rates for the INCA study do not show an increasing pattern as the model interval rates.

The overall program sensitivity was very similar (68.1% in the INCA study versus 68.4% in the mathematical model). By age group, both the INCA study and the mathematical model show an increasing trend (Table S12).

Table S13 presents the stages distribution for screen-detected and interval cases. Screen-detected cases in the model had a higher distribution of cases in Stages I and II than the INCA study,

which had a higher distribution of Stage III cases. Similarly, interval cases in the INCA study had a higher proportion of Stages III and IV cases. In summary, the stage distributions of the model, either screen-detected or interval, were more favorable than the cases in the INCA study.

G.2 Sensitivity analysis

G.2.1 Changes in the risk groups distribution

Figures S6 and S7 present the cost-effectiveness and harm-benefit analyses when 20% of the women in the L, ML, and MH risk groups migrated to the next higher risk group, for the selected risk-based strategies compared to the uniform B5069 and B4574 ones. With the new risk groups distribution, the selected risk-based strategies even perform better than with the former risk groups distribution.

G.2.2 Changes in the overdiagnosis rate of invasive tumors.

Tables S14 and S15 present the results obtained, for the benefit measures LE and QALY, respectively, when the assumptions on overdiagnosis rates for invasive BC and DCIS and the cost of cancer treatment were changed. In Table S14 the absolute values correspond to the selected Q5074-Q5074-T5074-A5074 strategy whereas in Table S15 they correspond to the selected Q5069-Q4574-Q4574-A4074 strategy. In both tables the percentages in parentheses correspond to relative changes with respect to the fixed B5069 strategy.

In the main analysis an overdiagnosis rate of 15% was assumed for the invasive tumors when the mammography exams were performed. The sensitivity analysis showed that, in absolute terms, the measures of benefit (LE and QALY) decreased when the overdiagnosis rate increased from 0% to 5% and from 5% to 15%. When the overdiagnosis rate increased from 15% to 25% the measures of benefit (LE and QALY) increased for the less intensive strategies and decreased for the more intensive strategies. The cost-benefit and the harm-benefit analyses were robust to changes in the overdiagnosis of invasive tumor rates. The resultant optimal strategies were mostly the same as in the main analysis but we observed changes in the incremental cost-benefit or harm-benefit ratios. Changes were more marked when the overdiagnosis rate increased from 15% to 25% where some less intensive screening strategies exited the Pareto frontier. In general, when the overdiagnosis rate increased, the incremental cost- or harm-benefit ratios also increased which

means that the cost or the harm for each additional unit of benefit was higher.

For the risk-based Q5074-Q5074-T5074-A5074 strategy, increasing the overdiagnosis rate of invasive tumors led to a decrease in the number of LE (Table S14), but the percentage change with respect to the fixed B5069 strategy increased. Similarly, for the risk-based Q5069-Q4574-Q4574-A4074 strategy, increasing the overdiagnosis rate of invasive tumors led to a decrease in the number of QALYs (Table S15) but, compared to the fixed B5069 strategy, the percentage change also increased.

G.2.3 Changes in the overdiagnosis rate of DCIS.

The overdiagnosis rate of DCIS is directly associated with a slight increase in costs (Tables S14 and S15) and a slight decrease in QALYs (Table S15). Since we assumed that overdiagnosed DCIS did not die of breast cancer, both LE and QALY were not affected by changes in the overdiagnosis rate. The resultant optimal strategies were the same as for the main analysis, except for one of the optimal strategies in the FN-QALY harm-benefit analysis.

For the risk-based Q5074-Q5074-T5074-A5074 and Q5069-Q4574-Q4574-A4074 strategies, increasing the overdiagnosis rate of DCIS led to an increase in costs and in the percentage change with respect to the fixed B5069 strategy (Tables S14 and S15).

G.2.4 Changes in the costs of breast cancer treatment.

When treatment costs increase, a reduced number of the strategies located in the left part of the frontier were not optimal anymore. This phenomenon was common to both benefit measures (LE and QALY) and was more marked for a 5-fold than for a 2-fold increase.

G.2.5 Changes in the disutility of FP results.

This sensitivity analysis only has an impact on QALYs. Increasing the disutility of FP results led to a decrease in QALY (Table S15).

Tables

Table S1: Distribution of stages at diagnosis of BC

	Stages[1]				
Age (years)	I	II-	II+	III	IV
Background[1,2]					
40-49	0.3008	0.2277	0.3091	0.0999	0.0625
50-59	0.2868	0.2176	0.3111	0.1021	0.0825
60-69	0.3028	0.2225	0.2713	0.0974	0.1061
70-79	0.3157	0.2671	0.2227	0.0983	0.0961
Annual screening. Screen-detected cases[1,3]					
40-49	0.6200	0.1131	0.2141	0.0436	0.0092
50-59	0.6669	0.1057	0.1935	0.0296	0.0043
60-69	0.7641	0.0739	0.1412	0.016	0.0047
70-79	0.7821	0.0875	0.1067	0.0165	0.0072
Annual screening. Interval cases[1,3]					
40-49	0.4644	0.1903	0.2598	0.0667	0.0188
50-59	0.4501	0.1744	0.2976	0.0665	0.0113
60-69	0.5417	0.1532	0.2320	0.0591	0.0141
70-79	0.5446	0.2345	0.1583	0.0496	0.013
Biennial screening. Screen-detected cases[1,3]					
40-49	0.5839	0.1217	0.2360	0.0438	0.0146
50-59	0.6210	0.1472	0.1734	0.0423	0.0161
60-69	0.6563	0.1295	0.1830	0.0246	0.0067
70-79	0.7287	0.1311	0.1128	0.0137	0.0137
Biennial screening. Interval cases[1,3]					
40-49	0.3673	0.2246	0.3099	0.0819	0.0164
50-59	0.2945	0.2609	0.2648	0.1166	0.0632
60-69	0.4077	0.2231	0.2672	0.0744	0.0275
70-79	0.4336	0.2885	0.1770	0.0673	0.0336
Triennial or quinquennial screening. Screen-detected cases[1,3]					
40-49	0.4910	0.1576	0.2605	0.0614	0.0295
50-59	0.5096	0.1707	0.2193	0.0622	0.0382
60-69	0.5384	0.1605	0.2124	0.0489	0.0398
70-79	0.5910	0.1765	0.1494	0.0419	0.0412
Triennial or quinquennial screening. Interval cases[1,3]					
40-49	0.3466	0.2262	0.3097	0.0868	0.0307
50-59	0.2919	0.2465	0.2802	0.1118	0.0696
60-69	0.3727	0.2229	0.2686	0.0821	0.0537
70-79	0.3943	0.2814	0.1922	0.0776	0.0545

[1] American Joint Committee on Cancer (AJCC) stage distribution.
[2] From Surveillance, Epidemiology, and End Results (SEER).
[3] From Breast Cancer Surveillance Consortium (BCSC).

Table S2: Relative risk of breast cancer based on age and breast density (the reference group for each relative risk is all women of that age group)[1]

BI-RADS[2] category	40-49 years	50-59 years	60-64 years	65-69 years	70-79 years
1	0.351	0.388	0.400	0.581	0.600
2	0.730	0.807	0.832	0.885	0.914
3	1.131	1.251	1.291	1.228	1.268
4	1.468	1.623	1.675	1.283	1.325

[1] From Schousboe *et al.* [10].
[2] BI-RADS (American College of Radiology Breast Imaging Reporting and Data System) 1 = almost entirely fat; 2 = scattered fibroglandular densities; 3 = heterogeneously dense; 4 = extremely dense.

Table S3: Prevalences of risk factors by age group for each category of breast density[1]

	35-39	40-44	45-49	50-54	55-59	60-64	65-69	70-74	75-79	80-84
BI-RADS[2]1										
0 RF[3]	0.0251	0.0343	0.0403	0.0521	0.0644	0.0778	0.0859	0.0920	0.0932	0.0943
1 RF[3]	0.0128	0.0078	0.0101	0.0143	0.0198	0.0241	0.0294	0.0330	0.0341	0.0346
2 RF[3]	0.0011	0.0006	0.0009	0.0015	0.0023	0.0030	0.0040	0.0036	0.0045	0.0042
BI-RADS[2]2										
0 RF[3]	0.1931	0.2604	0.2746	0.3089	0.3336	0.3461	0.3477	0.3490	0.3512	0.3501
1 RF[3]	0.1182	0.0699	0.0825	0.1016	0.1234	0.1386	0.1529	0.1626	0.1594	0.1581
2 RF[3]	0.0136	0.0062	0.0083	0.0120	0.0164	0.0198	0.0211	0.0233	0.0248	0.0246
BI-RADS[2]3										
0 RF[3]	0.2589	0.3452	0.3226	0.2850	0.2448	0.2149	0.1946	0.1812	0.1792	0.1806
1 RF[3]	0.1826	0.1109	0.1191	0.1216	0.1201	0.1144	0.1098	0.1051	0.1015	0.0974
2 RF[3]	0.0225	0.0111	0.0150	0.0168	0.0178	0.0170	0.0173	0.0168	0.0180	0.0173
BI-RADS[2]4										
0 RF[3]	0.0904	0.1060	0.0826	0.0537	0.0341	0.0258	0.0220	0.0194	0.0200	0.0241
1 RF[3]	0.0719	0.0424	0.0384	0.0285	0.0202	0.0160	0.0132	0.0117	0.0118	0.0125
2 RF[3]	0.0097	0.0051	0.0056	0.0039	0.0033	0.0023	0.0021	0.0022	0.0024	0.0023

[1] Based on the Risk Estimation Dataset of the Breast Cancer Surveillance Consortium (BCSC) [11].
[2] BI-RADS (American College of Radiology Breast Imaging Reporting and Data System) 1 = almost entirely fat; 2 = scattered fibroglandular densities; 3 = heterogeneously dense; 4 = extremely dense.
[3] RF = Risk Factor(s).

Table S4: Characteristics of the 2,625 screening strategies analized

Strategy	Interval between exams according to risk group years				Age at starting according to risk group years				Age at ending according to risk group years			
	L[1]	M-L[1]	M-H[1]	H[1]	L[1]	M-L[1]	M-H[1]	H[1]	L[1]	M-L[1]	M-H[1]	H[1]
1	5	5	5	5	50	50	50	50	69	69	69	69
2	5	5	5	5	50	50	50	50	69	69	69	74
3	5	5	5	5	50	50	50	50	69	69	74	74
4	5	5	5	5	50	50	50	50	69	74	74	74
5	5	5	5	5	50	50	50	50	74	74	74	74
6	5	5	5	5	50	50	50	45	74	74	74	74
...	...	...	...	...	...	...	...	...	...	...	...	...
...	5	3	2	1	50	50	45	40	69	69	74	74
...	...	...	...	...	...	...	...	...	...	...	...	...
2625	1	1	1	1	40	40	40	40	74	74	74	74

[1] L = Low risk; M-L = Medium-Low risk; M-H = Medium-High risk; H = High risk.

Table S5: The utilities for the general population and for women diagnosed with BC, either DCIS or invasive[1]

Age group	Women in the general population	DCIS first year	Local first year	Regional or distant first year	L[2], R[2] or D[2] successive years[3]	Four years before death
40-49	0.859	0.777	0.726	0.647	0.813	0.715
50-59	0.845	0.764	0.715	0.637	0.800	0.703
60-69	0.812	0.734	0.687	0.612	0.769	0.676
70-79	0.788	0.712	0.666	0.594	0.746	0.656

[1] Adapted from Lidgren *et al.* [12] and Schousboe *et al.* [10].
[2] L = local, R = regional, D = distant.
[3] QALY for DCIS in successive years are the same as for the general population.

Table S6: Model for false positive results of non-invasive tests

Variables	Coefficient	SE	p-value
Intercept	-1.2905	0.1857	<0.001
Screening round[1]	-0.1424	0.0160	<0.001
Age at screening	-0.0322	0.0031	<0.001
First screening round (*yes* versus *no*)	0.5520	0.0653	<0.001

[1] ranges from 1 to 6.

Table S7: Model for false positive results of invasive tests

Variables	Coefficient	SE	p-value
Intercept	-4.6539	0.1878	<0.001
Screening round[1]	-0.1483	0.0162	<0.001
Age at screening	-0.0195	0.0032	<0.001
First screening round (*yes* versus *no*)	1.0638	0.0660	<0.001

[1] ranges from 1 to 6.

Table S8: Distribution of stages at diagnosis of BC for screen-detected cases. Different overdiagnosis rates[1]

	Stages				
Age (years)	I	II-	II+	III	IV
Annual screening					
Overdiagnosis rate 5%					
40-49	0.6000	0.1191	0.2254	0.0459	0.0097
50-59	0.6494	0.1113	0.2037	0.0312	0.0045
60-69	0.7517	0.0778	0.1486	0.0168	0.0049
70-79	0.7706	0.0921	0.1123	0.0174	0.0076
Overdiagnosis rate 15%					
40-49	0.5529	0.1331	0.2519	0.0513	0.0108
50-59	0.6081	0.1244	0.2276	0.0348	0.0051
60-69	0.7225	0.0869	0.1661	0.0188	0.0055
70-79	0.7436	0.1029	0.1255	0.0194	0.0085
Overdiagnosis rate 25%					
40-49	0.4933	0.1508	0.2855	0.0581	0.0123
50-59	0.5559	0.1409	0.2580	0.0395	0.0057
60-69	0.6855	0.0985	0.1883	0.0213	0.0063
70-79	0.7095	0.1167	0.1423	0.0220	0.0096
Biennial screening					
Overdiagnosis rate 5%					
40-49	0.5620	0.1281	0.2484	0.0461	0.0154
50-59	0.6011	0.1549	0.1825	0.0445	0.0169
60-69	0.6382	0.1363	0.1926	0.0259	0.0071
70-79	0.7144	0.1380	0.1187	0.0144	0.0144
Overdiagnosis rate 15%					
40-49	0.5105	0.1432	0.2776	0.0515	0.0172
50-59	0.5541	0.1732	0.2040	0.0498	0.0189
60-69	0.5956	0.1524	0.2153	0.0289	0.0079
70-79	0.6808	0.1542	0.1327	0.0161	0.0161
Overdiagnosis rate 25%					
40-49	0.4452	0.1623	0.3147	0.0584	0.0195
50-59	0.4947	0.1963	0.2312	0.0564	0.0215
60-69	0.5417	0.1727	0.2440	0.0328	0.0089
70-79	0.6383	0.1748	0.1504	0.0183	0.0183
Triennial or quinquennial screening					
Overdiagnosis rate 5%					
40-49	0.4642	0.1659	0.2742	0.0646	0.0311
50-59	0.4838	0.1797	0.2308	0.0655	0.0402
60-69	0.5141	0.1689	0.2236	0.0515	0.0419
70-79	0.5695	0.1858	0.1573	0.0441	0.0434
Overdiagnosis rate 15%					
40-49	0.4012	0.1854	0.3065	0.0722	0.0347
50-59	0.4231	0.2008	0.2580	0.0732	0.0449
60-69	0.4569	0.1888	0.2499	0.0575	0.0468
70-79	0.5188	0.2076	0.1758	0.0493	0.0485
Overdiagnosis rate 25%					
40-49	0.3213	0.2101	0.3473	0.0819	0.0393
50-59	0.3461	0.2276	0.2924	0.0829	0.0509
60-69	0.3845	0.2140	0.2832	0.0652	0.0531
70-79	0.4547	0.2353	0.1992	0.0559	0.0549

[1] Distribution of stages at diagnosis of BC for screen-detected cases with an overdiagnosis rate of 0%, same as Table S1.

Table S9: Linear regression model with dependent variable being the DCIS rate per 10^5 women-year

Variables	Coefficient	SE	p-value
Intercept	0.4027	1.9045	0.838
Age group 50-69 years[1]	2.6302	1.9898	0.223
Age group $\geq$70 years[1]	2.8019	2.1886	0.236
Index of mammography usage (IMU)[2]	31.1336	4.5488	< 0.001

R^2=0.87

[1] The reference group for the variable age group is 40-49 years.

[2] Mammogram usage is an index that measures the intensity of mammography use per woman-year. It takes values between 0 and 1. If nobody is using mammography for early detection IMU=0. If every woman in the population receives an annual mammography, IMU=1. If every woman receives a biennial mammography, IMU=0.5.

Table S10: Cost-effectiveness and harm-benefit analysis. Lives extended (LE)[1]

Cost-effectiveness analysis. Incremental cost per incremental LE, per 100,000 women at birth

Strategy	LE	Cost ($\times 10^6$euros)	ΔLE	ΔCost ($\times 10^6$ euros)	ICER euros/LE
Q5069-Q5069-Q5069-Q5074	146.6	123.8			
Q5069-Q5069-Q5074-Q5074	157.8	123.9	11.3	0.1	0.01
Q5069-Q5074-Q5074-Q5074	177.5	124.3	19.7	0.4	0.02
Q5074-Q5074-Q5074-Q5074	189.1	124.8	11.6	0.5	0.04
Q5074-Q5074-Q5074-Q4574	190.0	124.8	0.9	0.0	0.06
Q5074-Q5074-Q5074-T5074	191.5	125.0	1.6	0.1	0.09
Q5074-Q5074-Q5074-A5074	197.7	125.6	6.1	0.6	0.10
Q5074-Q5074-T5074-A5074	209.5	127.1	11.8	1.5	0.13
Q5074-Q5074-A5074-A5074	238.8	132.5	29.3	5.4	0.18
Q5074-T5074-A5074-A5074	258.7	136.7	19.9	4.2	0.21
Q5074-T5074-A5074-A4574	260.2	137.0	1.4	0.4	0.25
Q5074-A5074-A5074-A4574	309.5	152.8	49.3	15.7	0.32
T5074-A5074-A5074-A4574	320.4	156.6	10.9	3.9	0.35
T5074-A5074-A4574-A4574	327.2	159.7	6.8	3.1	0.45
T5074-A5074-A4574-A4074	328.2	160.2	1.0	0.5	0.46
A5074-A5074-A4574-A4074	354.9	175.4	26.7	15.2	0.57
A5074-A5074-A4074-A4074	359.7	179.3	4.8	3.9	0.80
A5074-A4574-A4074-A4074	370.7	188.1	11.0	8.8	0.80
A5074-A4074-A4074-A4074	378.6	198.9	7.8	10.8	1.38
A4574-A4074-A4074-A4074	384.3	207.3	5.7	8.4	1.46
A4074-A4074-A4074-A4074	388.4	217.4	4.1	10.2	2.47

Incremental false-positives (FP) per incremental LE, per 100,000 women at birth

Strategy	LE	FP	ΔLE	Δ FP	ΔFP/ΔLE
Q5069-Q5069-Q5069-Q5069	144.2	12,995.4			
Q5069-Q5069-Q5069-Q5074	146.6	13,006.2	2.3	10.8	4.7
Q5069-Q5069-Q5074-Q5074	157.8	13,090.8	11.3	84.5	7.5
Q5069-Q5074-Q5074-Q5074	177.5	13,321.9	19.7	231.2	11.7
Q5074-Q5074-Q5074-Q5074	189.1	13,536.1	11.6	214.2	18.5
Q5074-Q5074-Q5074-T5074	191.5	13,607.4	2.4	71.3	29.1
Q5074-Q5074-Q5074-A5074	197.7	13,856.0	6.1	248.5	40.5
Q5074-Q5074-T5074-A5074	209.5	14,415.2	11.8	559.3	47.5
Q5074-Q5074-A5074-A5074	238.8	16,363.8	29.3	1,948.6	66.4
Q5074-T5074-A5074-A5074	258.7	17,893.3	19.9	1,529.5	76.7
Q5074-T5074-A5074-A4574	260.2	18,034.1	1.4	140.8	97.2
Q5074-A5074-A5074-A4574	309.5	23,362.9	49.3	5,328.8	108.1
T5074-A5074-A5074-A4574	320.4	24,780.0	10.9	1,417.1	129.5
A5074-A5074-A5074-A4574	347.1	29,717.0	26.7	4,937.1	184.8
A5074-A5074-A4574-A4574	353.9	31,031.0	6.8	1,313.9	194.4
A5074-A5074-A4574-A4074	354.9	31,240.1	1.0	209.2	201.0
A5074-A4574-A4574-A4074	365.9	35,176.2	11.0	3,936.1	358.2
A5074-A4574-A4074-A4074	370.7	36,994.8	4.8	1,818.6	376.2
A4574-A4574-A4074-A4074	376.5	40,844.6	5.7	3,849.9	670.7
A4574-A4074-A4074-A4074	384.3	46,107.9	7.8	5,263.3	671.2
A4074-A4074-A4074-A4074	388.4	51,155.8	4.1	5,047.9	1,224.1

Incremental overdiagnosis (ODX) per incremental LE, per 100,000 women at birth					
Strategy	LE	ODX	ΔLE	ΔODX	ΔODX/ΔLE
Q5069-Q5069-Q5069-Q5069	144.2	187.2			
Q5069-Q5069-Q5069-Q5074	146.6	189.3	2.3	2.1	0.9
Q5069-Q5069-Q5074-Q5074	157.8	200.1	11.3	10.8	1.0
Q5069-Q5074-Q5074-Q5074	177.5	220.2	19.7	20.2	1.0
Q5074-Q5074-Q5074-Q5074	189.1	233.4	11.6	13.2	1.1
Q5074-Q5074-Q5074-Q4574	190.0	234.6	0.9	1.1	1.3
Q5074-Q5074-Q5074-Q4074	190.6	235.3	0.6	0.8	1.3
Q5074-Q5074-Q4574-Q4074	194.6	241.6	4.0	6.3	1.6
Q5074-Q5074-Q4074-Q4074	197.3	246.5	2.8	4.9	1.8
Q5074-Q5074-Q4574-A4574	203.1	257.6	5.8	11.0	1.9
Q5074-Q5074-A5074-A5074	238.8	326.9	35.7	69.3	1.9
Q5074-A5074-A5074-A5074	308.0	493.4	69.2	166.5	2.4
Q5074-A5074-A5074-A4574	309.5	497.2	1.4	3.8	2.6
Q5074-A5074-A5074-A4074	310.5	500.5	1.0	3.2	3.1
T5074-A5074-A5074-A4074	321.4	536.2	10.9	35.7	3.3
A5074-A5074-A5074-A4074	348.1	623.7	26.7	87.5	3.3
A5074-A5074-A4574-A4074	354.9	646.5	6.8	22.9	3.4
A5074-A5074-A4074-A4074	359.7	667.5	4.8	21.0	4.3
A5074-A4574-A4074-A4074	370.7	718.6	11.0	51.1	4.7
A5074-A4074-A4074-A4074	378.6	769.0	7.8	50.4	6.4
A4574-A4074-A4074-A4074	384.3	809.6	5.7	40.6	7.1
A4074-A4074-A4074-A4074	388.4	852.2	4.1	42.6	10.3
Incremental false-negatives (FN) per incremental LE, per 100,000 women at birth					
Strategy	LE	FN	ΔLE	ΔFN	ΔFN/ΔLE
A5069-A5069-A5069-A5069	297.1	158.9			
A5074-A5074-A5074-A5074	345.7	180.9	48.6	22.0	0.5
A5074-A5074-A5074-A4574	347.1	182.8	1.4	1.8	1.3
A5074-A5074-A4574-A4574	353.9	191.3	6.8	8.6	1.3
A5074-A4574-A4574-A4574	364.9	205.2	11.0	13.9	1.3
A4574-A4574-A4574-A4574	370.6	212.5	5.7	7.3	1.3
A4574-A4574-A4574-A4074	371.6	215.4	1.0	2.9	2.7
A4074-A4074-A4074-A4074	388.4	261.6	16.8	46.2	2.8

[1] Data corresponding to a cohort of 100,000 women at birth assessed in the age-interval 40-79 years. All the absolute values have been discounted at an annual rate of 3%.

Table S11: Cost-effectiveness and harm-benefit analysis. Quality-adjusted life years (QALY)[1]

Cost-effectiveness analysis. Incremental cost per incremental QALY, per 100,000 women at birth					
Schedule	QALY	Cost ($\times 10^6$ euros)	ΔQALY	ΔCost ($\times 10^6$ euros)	ΔCost/ΔQALY euros/QALY
Q5069-Q5069-Q5069-Q5074	1,816.3	123.8			
Q5069-Q5069-Q5074-Q5074	1,869.5	123.9	53.2	0.1	0.001
Q5069-Q5069-Q5074-Q4574	1,892.0	123.9	22.5	0.0	0.002
Q5069-Q5074-Q5074-Q4574	1,983.9	124.3	91.9	0.4	0.004
Q5069-Q5074-Q4574-Q4574	2,084.5	124.9	100.6	0.6	0.006
Q5069-Q5074-Q4574-Q4074	2,101.8	125.0	17.3	0.1	0.006
Q5074-Q5074-Q4574-Q4074	2,154.7	125.5	52.9	0.5	0.010
Q5074-Q5074-Q4574-A4574	2,241.0	126.5	86.3	1.0	0.012
Q5074-Q4574-Q4574-A4574	2,393.3	128.5	152.3	1.9	0.013
Q5074-Q4574-Q4074-A4074	2,496.3	129.9	103.0	1.4	0.014
Q5074-Q4574-A4574-A4074	2,893.2	138.3	396.8	8.4	0.021
Q4574-Q4574-A4574-A4074	2,961.9	140.3	68.8	2.0	0.029
Q4574-Q4074-A4074-A4074	3,187.2	146.9	225.2	6.6	0.029
Q4569-A4569-A4069-A4074	3,682.2	165.6	495.1	18.7	0.038
Q4574-A4574-A4074-A4074	3,810.9	171.0	128.7	5.3	0.042
A5069-A4569-A4074-A4074	3,987.3	181.2	176.4	10.2	0.058
A5069-A4069-A4074-A4074	4,148.0	192.0	160.7	10.8	0.067
A5069-A4074-A4074-A4074	4,193.8	195.6	45.8	3.6	0.078
A4569-A4074-A4074-A4074	4,286.7	203.9	92.9	8.4	0.090
A4574-A4074-A4074-A4074	4,307.6	207.3	20.9	3.3	0.159
A4074-A4074-A4074-A4074	4,360.5	217.4	52.9	10.2	0.192

Incremental false-positive (FP) per incremental QALY, per 100,000 women at birth					
Schedule	QALY	FP	ΔQALY	ΔFP	ΔFP/ΔQALY
Q5069-Q5069-Q5069-Q5069	1,805.3	12,995.4			
Q5069-Q5069-Q5069-Q5074	1,816.3	13,006.2	11.0	10.8	1.0
Q5069-Q5069-Q5069-Q4574	1,838.8	13,034.5	22.5	28.3	1.3
Q5069-Q5069-Q5074-Q4574	1,892.0	13,119.0	53.2	84.5	1.6
Q5069-Q5074-Q5074-Q4574	1,983.9	13,350.2	91.9	231.2	2.5
Q5074-Q5074-Q5074-Q4574	2,036.8	13,564.4	52.9	214.2	4.0
Q5074-Q5074-Q5074-A4574	2,140.4	13,996.8	103.6	432.4	4.2
Q5074-Q5074-Q4574-A4574	2,241.0	14,428.4	100.6	431.7	4.3
Q5074-Q5074-A5074-A4574	2,559.7	16,504.6	318.7	2,076.2	6.5
Q5074-Q5074-A5074-A4074	2,587.6	16,713.8	27.9	209.2	7.5
Q5074-Q5074-A4574-A4074	2,740.9	18,027.7	153.3	1,313.9	8.6
Q5074-Q4574-A4574-A4074	2,893.2	19,551.1	152.3	1,523.3	10.0
Q5069-A5069-A4569-A4074	3,271.0	23,362.4	377.8	3,811.4	10.1
Q5069-A5069-A4574-A4074	3,300.6	23,713.0	29.6	350.6	11.9
Q5074-A5074-A4574-A4074	3,399.3	24,886.0	98.8	1,173.0	11.9
Q5074-A5074-A4074-A4074	3,517.7	26,704.6	118.4	1,818.6	15.4
Q5074-A4574-A4074-A4074	3,742.2	30,640.6	224.5	3,936.1	17.5
A5069-A4569-A4074-A4074	3,987.3	35,147.7	245.1	4,507.0	18.4
A5069-A4574-A4074-A4074	4,033.1	36,106.5	45.8	958.8	20.9
A5069-A4074-A4074-A4074	4,193.8	41,369.7	160.6	5,263.3	32.8
A4569-A4074-A4074-A4074	4,286.7	45,219.6	92.9	3,849.9	41.5
A4574-A4074-A4074-A4074	4,307.6	46,107.9	20.9	888.3	42.5
A4074-A4074-A4074-A4074	4,360.5	51,155.8	52.9	5,047.9	95.3
**Incremental overdiagnosis (ODX) per incremental QALY, per 100,000 women at birth**					
Schedule	QALY	ODX	ΔQALY	ΔODX	ΔODX/ΔQALY
Q5069-Q5069-Q5069-Q5069	1,805.3	187.2			
Q5069-Q5069-Q5069-Q4069	1,845.1	189.1	39.8	1.9	0.05
Q5069-Q5069-Q4569-Q4069	1,945.6	195.3	100.4	6.3	0.06
Q5069-Q5069-Q4069-Q4069	2,020.7	200.3	75.1	4.9	0.07
Q5069-Q4569-Q4069-Q4069	2,172.6	213.4	151.9	13.2	0.09
Q5069-Q4069-Q4069-Q4069	2,279.6	224.9	106.9	11.5	0.11
Q5069-Q4069-Q4069-A4069	2,398.3	241.8	118.7	16.9	0.14
Q4569-Q4069-Q4069-A4069	2,466.7	251.6	68.4	9.8	0.14
Q4569-Q4069-A4069-A4069	3,005.6	351.1	539.0	99.5	0.18
Q4069-Q4069-A4069-A4069	3,046.9	360.5	41.3	9.4	0.23
Q4569-A4569-A4069-A4069	3,675.8	518.5	628.8	158.0	0.25
Q4069-A4069-A4069-A4069	3,877.7	578.4	201.9	59.9	0.30
A5069-A4069-A4069-A4069	4,111.9	664.4	234.3	86.1	0.37
A4569-A4069-A4069-A4069	4,204.8	705.0	92.9	40.6	0.44
A4569-A4069-A4069-A4074	4,211.3	709.4	6.5	4.4	0.68
A4569-A4069-A4074-A4074	4,240.9	732.8	29.6	23.4	0.79
A4069-A4069-A4074-A4074	4,293.8	775.4	52.9	42.6	0.80
A4069-A4074-A4074-A4074	4,339.6	821.1	45.8	45.7	1.00
A4074-A4074-A4074-A4074	4,360.5	852.2	20.9	31.1	1.49
**Incremental false-negatives (FN) per incremental QALY, per 100,000 women at birth**					
Schedule	QALY	FN	ΔQALY	ΔFN	ΔFN/ΔQALY
A5069-A5069-A5069-A5069	3,392.5	158.9			
A5069-A5069-A5069-A4569	3,427.3	160.7	34.8	1.8	0.05
A5069-A5069-A4569-A4569	3,580.5	169.3	153.3	8.6	0.06
A5069-A4569-A4569-A4569	3,805.0	183.2	224.5	13.9	0.06
A4569-A4569-A4569-A4569	3,897.9	190.5	92.9	7.3	0.08
A4569-A4569-A4569-A4069	3,925.8	193.3	27.9	2.9	0.10
A4569-A4569-A4069-A4069	4,044.2	206.7	118.4	13.3	0.11
A4569-A4069-A4069-A4069	4,204.8	228.3	160.7	21.6	0.13
A4569-A4069-A4069-A4074	4,211.3	229.5	6.5	1.2	0.19
A4569-A4069-A4074-A4074	4,240.9	235.4	29.6	5.9	0.20
A4069-A4069-A4074-A4074	4,293.8	246.7	52.9	11.3	0.21
A4069-A4074-A4074-A4074	4,339.6	256.5	45.8	9.8	0.21
A4074-A4074-A4074-A4074	4,360.5	261.6	20.9	5.1	0.24

[1] Data corresponding to a cohort of 100,000 women at birth assessed in the age-interval 40-79 years. All the absolute values have been discounted at an annual rate of 3%.

Table S12: Number of mammograms and detection rates for screen-detected and interval cases and program sensitivity by age groups. Invasive cancer (DCIS not included).

	Number of mammograms	Screen-detected		Interval cancer		Program sensitivity
	N	N	‰	N	‰	%
			The INCA study			
Total	1,508,584	3,096	2.05	1,448	0.96	68.13
44-49	65,177	168	2.58	74	1.14	69.42
50-54	480,094	808	1.68	512	1.07	61.21
55-59	441,633	808	1.83	400	0.91	66.89
60-64	383,641	860	2.24	325	0.85	72.57
65-70	138,039	452	3.27	137	1.00	76.74
			Mathematical model[1], biennial screening			
Total	1,186,681	3,021	2.55	1,399	1.18	68.35
45-49	235,524	297	1.26	192	0.82	60.74
50-54	233,102	460	1.97	251	1.08	64.70
55-59	229,681	555	2.42	257	1.12	68.35
60-64	225,542	669	2.97	352	1.56	65.52
65-70	262,832	1,040	3.96	347	1.32	74.98

[1] The results correspond to the fixed B4569 strategy.

Table S13: Distribution of stages at diagnosis of BC

Age (years)	Stages I %	II %	III %	IV %
Screen-detected cases. INCA study				
44-49	64.7	23.5	9.8	2.0
50-59	59.0	31.3	8.9	0.8
60-69	64.9	26.3	8.0	0.8
Interval cases. INCA study				
44-49	30.6	45.2	21.0	3.2
50-59	27.5	43.8	24.8	4.0
60-69	28.5	46.1	20.9	4.5
Screen-detected cases. Model inputs[1]				
40-49	58.4	35.8	4.4	1.5
50-59	62.1	32.1	4.2	1.6
60-69	65.6	31.3	2.5	0.7
Interval cases. Model inputs[1]				
40-49	36.7	53.5	8.2	1.6
50-59	29.5	52.6	11.7	6.3
60-69	40.8	49.0	7.4	2.8

[1] The results correspond to the fixed B4569 strategy.

Table S14: Sensitivity analysis. Changes in lives extended[1,2]

Overdiagnosis rate invasive tumors	Overdiagnosis rate (per 1,000) of DCIS	Changes in costs	LE N (%)	Cost N (%)	Overdiagnosis N (%)
0%	-	-	228.5 (1.1)	126.1 (-8.8)	63.6 (-42.5)
5%	-	-	222.8 (1.8)	126.4 (-8.9)	134.4 (-29.1)
-	0.10	-	209.5 (3.8)	126.6 (-8.7)	242.6 (-16.2)
15%	0.21	C[3]	209.5 (3.8)	127.1 (-8.9)	275.9 (-20.6)
-	-	2 times C	209.5 (3.8)	219.4 (-5.6)	275.9 (-20.6)
-	-	5 times C	209.5 (3.8)	496.2 (-2.9)	275.9 (-20.6)
-	0.26	-	209.5 (3.8)	127.4 (-9.0)	291.1 (-22.1)
25%	-	-	192.5 (6.7)	128.1 (-9.0)	417.5 (-17.4)

[1] Data corresponding to a cohort of 100,000 women at birth assessed in the age-interval 40-79 years. All the absolute values have been discounted at an annual rate of 3%.
[2] Results obtained for the risk-based strategy Q5074-Q5074-T5074-A5074, and the percentages of change compared to the B5069 fixed strategy.
[3] C: costs in the main analysis.

Table S15: Sensitivity analysis. Changes in QALY[1,2]

Overdiagnosis rate invasive tumors	Overdiagnosis rate (per 1,000) of DCIS	Changes in costs	Changes in disutility by FP	QALY N (%)	Cost N (%)	Overdiagnosis N (%)
0%	-	-	-	2616.3 (-1.0)	127.4 (-7.8)	68.3 (-38.2)
5%	-	-	-	2542.4 (-0.3)	127.7 (-7.9)	132.4 (-30.1)
-	0.10	-	-	2371.2 (1.4)	127.9 (-7.8)	224.9 (-22.3)
-	-	-	0 times D[4]	2492.5 (-0.5)	128.4 (-8.0)	260.7 (-25.0)
15%	0.21	C[3]	D	2368.3 (1.5)	128.4 (-8.0)	260.7 (-25.0)
-	-	-	2 times D	2244.1 (3.8)	128.4 (-8.0)	260.7 (-25.0)
-	-	2 times C	-	2368.3 (1.5)	220.7 (-5.0)	260.7 (-25.0)
-	-	5 times C	-	2368.3 (1.5)	497.4 (-2.6)	260.7 (-25.0)
-	0.26	-	-	2367.0 (1.5)	128.7 (-8.1)	277.0 (-25.9)
25%	-	-	-	2147.4 (4.3)	129.3 (-8.1)	389.0 (-23.1)

[1] Data corresponding to a cohort of 100,000 women at birth assessed in the age-interval 40-79 years. All the absolute values have been discounted at an annual rate of 3%.

[2] Results obtained for the risk-based strategy Q5069-Q4574-Q4574-A4074, and the percentages of change compared to the B5069 fixed strategy.

[3] C: costs in the main analysis.

[4] D: disutility in the main analysis.

Figures

Figure S1: Incidence curves for twelve risk profiles grouped by risk level: (A) Low Risk, (B) Medium-Low Risk, (C) Medium-High Risk, and (D) High Risk. Graphic (E) shows the smoothed incidence rates for each risk group.

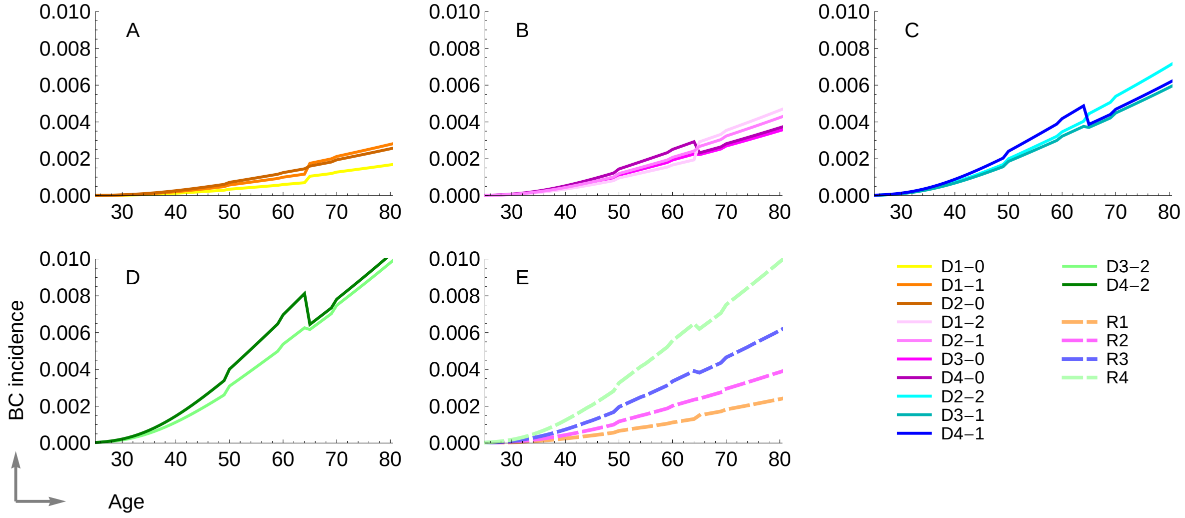

Based on Schousboe *et al.* [10], Tice *et al.* [9], and the Risk Estimation Dataset of the Breast Cancer Surveillance Consortium (BCSC) [11].

Figure S2: Observed and smoothed DCIS rates over time in Catalonia (1983-2008)

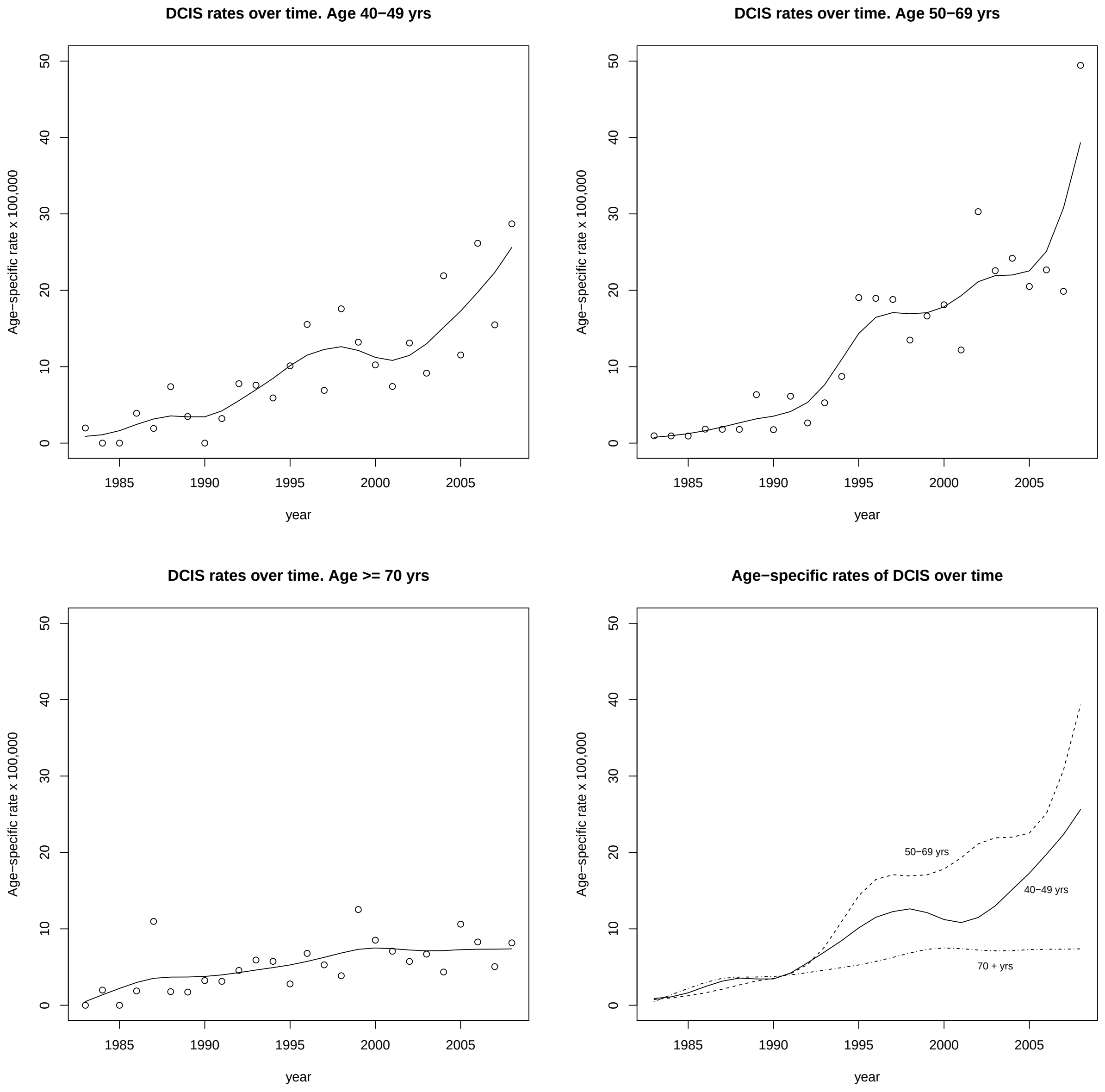

Figure S3: Index of mammography use (IMU) and smoothed DCIS rates over time in Catalonia (1983-2008)

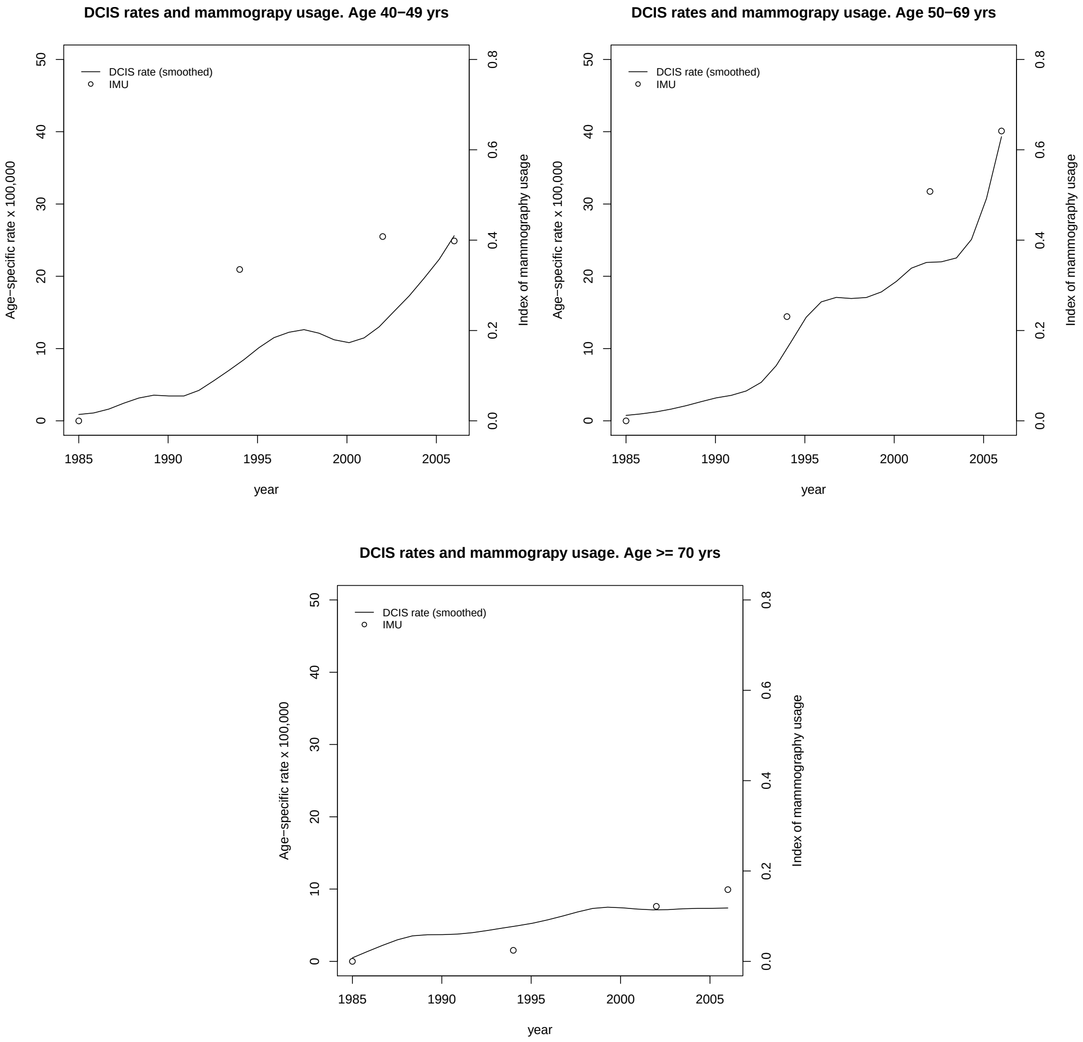

Figure S4: Cost-effectiveness and harm-benefit analyses for 2,625 early detection strategies, with uniform strategies marked. Effect measured in lives extended

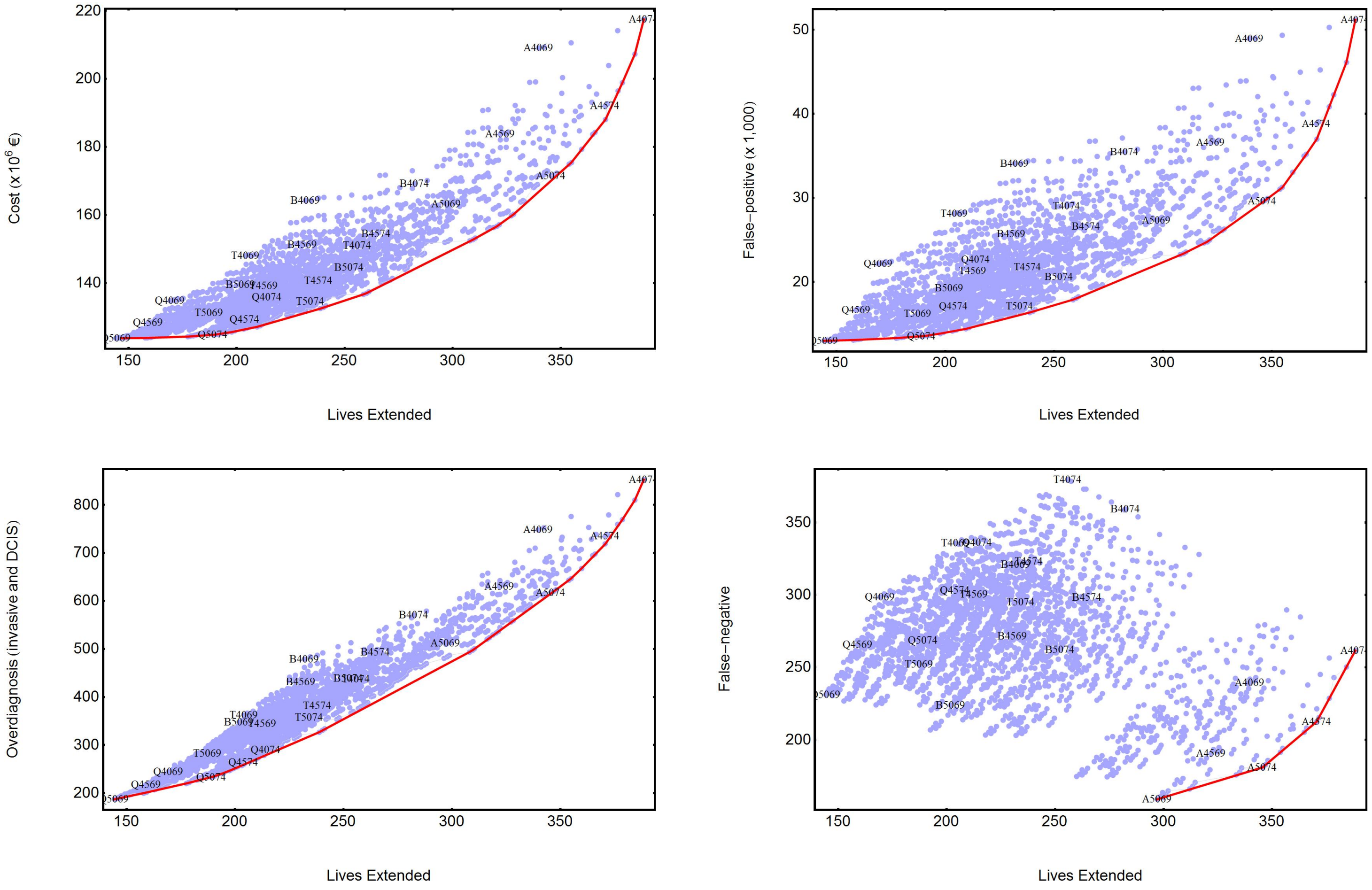

Results obtained with an annual discount of 3%. Exams periodicities: A = annual, B = biennial, T = triennial, Q = quinquennial. The first two numbers refer to the age at starting the exams and the last two numbers refer to the age at the last exam.

Figure S5: Cost-effectiveness and harm-benefit analyses for 2,625 early detection strategies, with uniform strategies marked. Effect measured in quality-adjusted life years

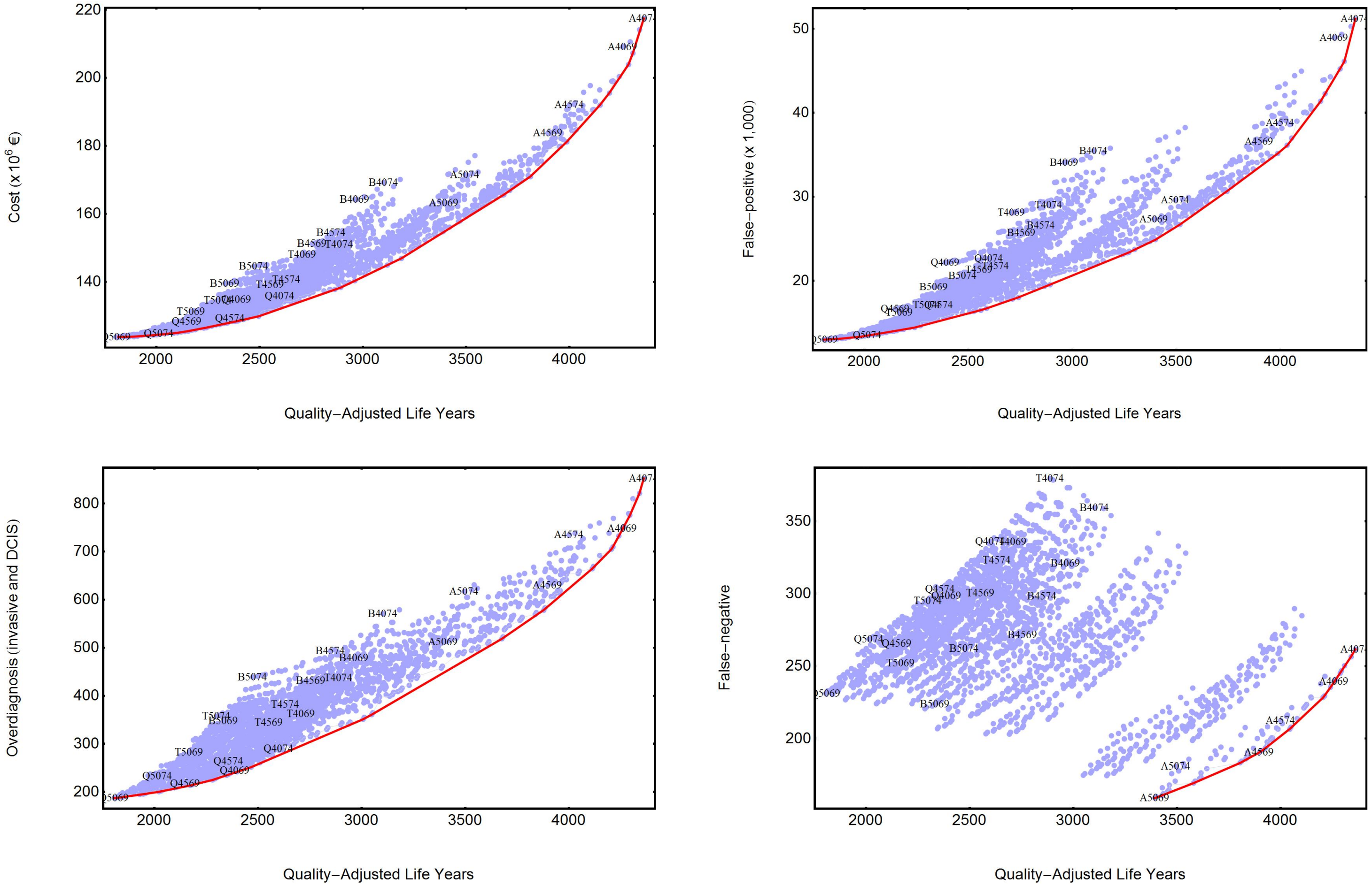

Results obtained with an annual discount of 3%. Exams periodicities: A = annual, B = biennial, T = triennial, Q = quinquennial.The first two numbers refer to the age at starting the exams and the last two numbers refer to the age at the last exam.

Figure S6: Sensitivity analysis of a change in the risk groups distribution. Cost-effectiveness and harm-benefit analyses for 2,625 early detection strategies. Effect measured in lives extended

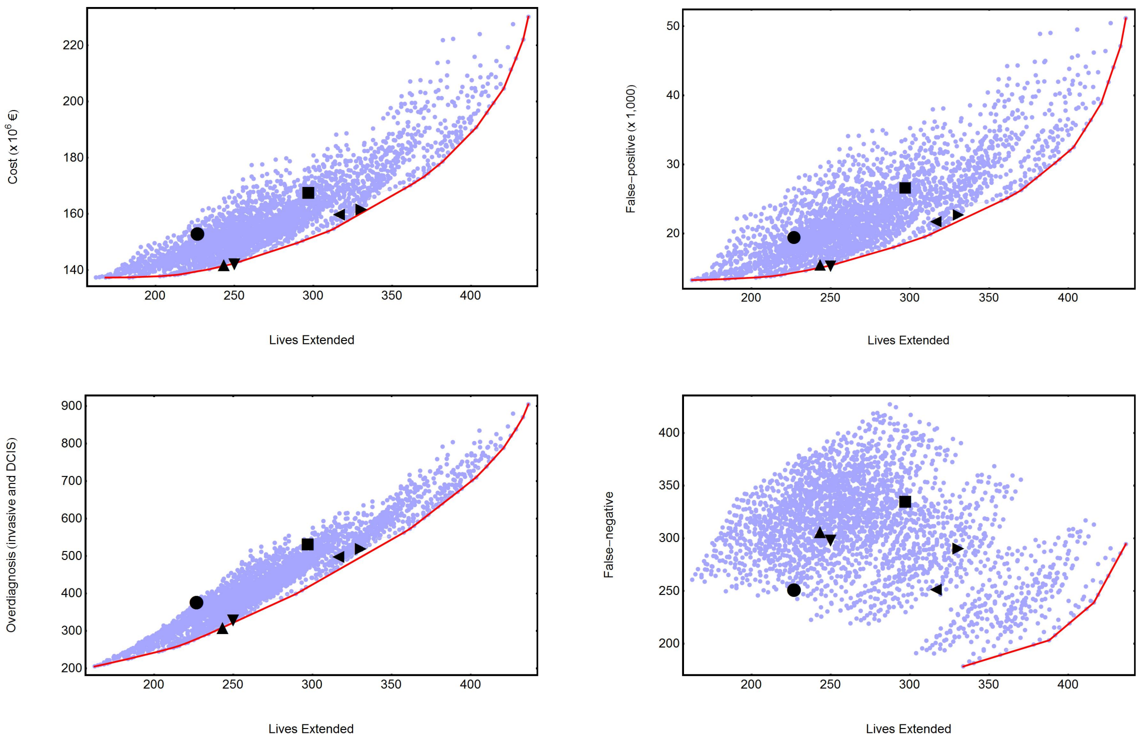

●: uniform B5069; ■: uniform B4574

▲: risk-based Q5074-Q5074-Q4574-A4574; ▼: risk-based Q5074-Q5074-T5074-A5074

◀: risk-based T5069-B5074-A5074-A5074; ▶: risk-based T5074-T5074-A4574-A4574

Results obtained with an annual discount of 3%.

Exams periodicities: A = annual, B = biennial, T = triennial, Q = quinquennial.

The first two numbers refer to the age at starting the exams and the last two numbers refer to the age at the last exam. In the risk-based strategies, the four strings correspond to the Low, Medium-Low, Medium-High and High risk groups, respectively.

Figure S7: Sensitivity analysis of a change in the risk groups distribution. Cost-effectiveness and harm-benefit analyses for 2,625 early detection strategies. Effect measured in quality-adjusted life years

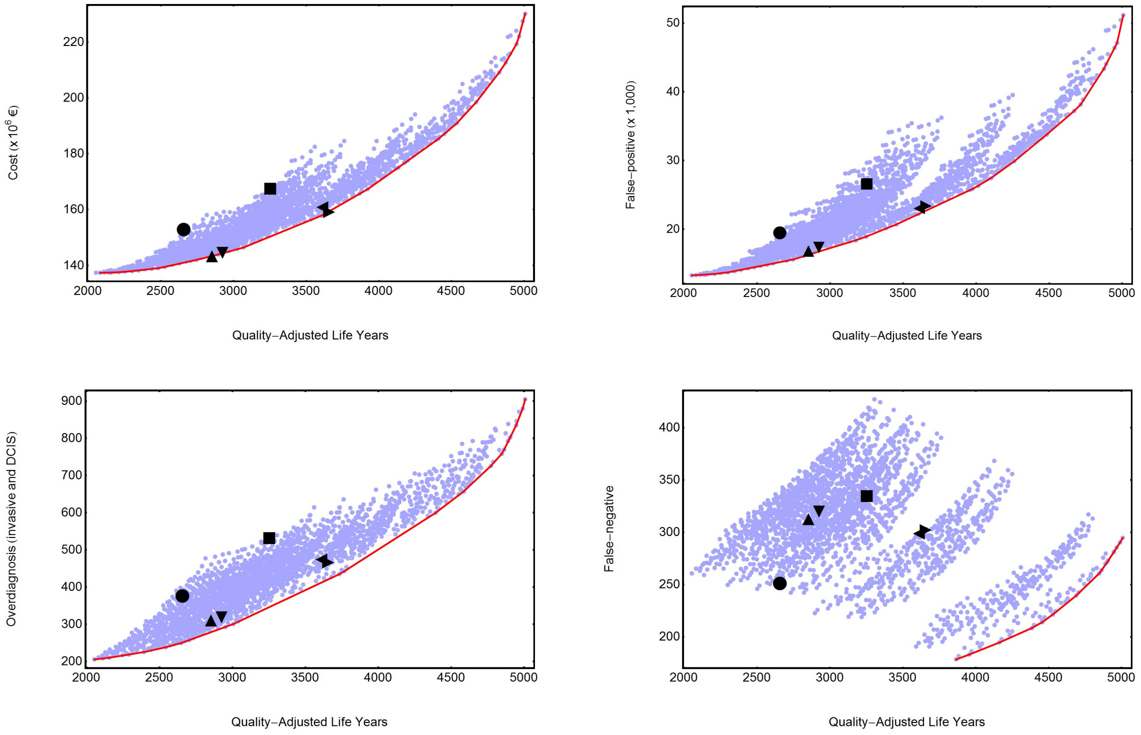

●: uniform B5069; ■: uniform B4574
▲: risk-based Q5069-Q4574-Q4574-A4574; ▼: risk-based Q5069-Q4574-Q4574-A4074
◀: risk-based Q5074-Q5074-A4074-A4074; ▶: risk-based Q4574-Q4574-A4574-A4074
Results obtained with an annual discount of 3%.
Exams periodicities: A = annual, B = biennial, T = triennial, Q = quinquennial.
The first two numbers refer to the age at starting the exams and the last two numbers refer to the age at the last exam. In the risk-based strategies, the four strings correspond to the Low, Medium-Low, Medium-High and High risk groups, respectively.

References

1. Lee S, Zelen M (1998) Scheduling periodic examinations for the early detection of disease: applications to breast cancer. J Am Stat Assoc (93): 1271-1281.

2. Lee S, Zelen M (2006) A stochastic model for predicting the mortality of breast cancer. J Natl Cancer Inst Monogr (36): 79-86.

3. Lee SJ, Zelen M (2008) Mortality modeling of early detection programs. Biometrics 64: 386-395.

4. Vilaprinyo E, Rue M, Marcos-Gragera R, Martinez-Alonso M (2009) Estimation of age- and stage-specific Catalan breast cancer survival functions using US and Catalan survival data. BMC Cancer 9: 98, doi:10.1186/1471-2407-9-98.

5. Mariotto A, Feuer EJ, Harlan LC, Wun LM, Johnson KA, et al. (2002) Trends in use of adjuvant multi-agent chemotherapy and tamoxifen for breast cancer in the United States: 1975-1999. J Natl Cancer Inst 94: 1626-1634.

6. Chia SK, Speers CH, D'yachkova Y, Kang A, Malfair-Taylor S, et al. (2007) The impact of new chemotherapeutic and hormone agents on survival in a population-based cohort of women with metastatic breast cancer. Cancer 110: 973-979.

7. Martinez-Alonso M, Vilaprinyo E, Marcos-Gragera R, Rue M (2010) Breast cancer incidence and overdiagnosis in Catalonia (Spain). Breast Cancer Res 12: R58.

8. American College of Radiology (2003) The American College of Radiology Breast Imaging Reporting and Data System (BI-RADS). American College of Radiology, Reston (VA).

9. Tice JA, Cummings SR, Smith-Bindman R, Ichikawa L, Barlow WE, et al. (2008) Using clinical factors and mammographic breast density to estimate breast cancer risk: development and validation of a new predictive model. Ann Intern Med 148: 337-347.

10. Schousboe JT, Kerlikowske K, Loh A, Cummings SR (2011) Personalizing mammography by breast density and other risk factors for breast cancer: analysis of health benefits and cost-effectiveness. Ann Intern Med 155: 10-20.

11. Barlow WE, White E, Ballard-Barbash R, Vacek PM, Titus-Ernstoff L, et al. (2006) Prospective breast cancer risk prediction model for women undergoing screening mammography. J Natl Cancer Inst 98: 1204-1214.

12. Lidgren M, Wilking N, Jonsson B, Rehnberg C (2007) Health related quality of life in different states of breast cancer. Qual Life Res 16: 1073-1081.

13. Rue M, Carles M, Vilaprinyo E, Martinez-Alonso M, Espinas JA, et al. (2008) Dissemination of periodic mammography and patterns of use, by birth cohort, in Catalonia (Spain). BMC Cancer 8: 336.

14. Perez MJ, Gregori A, Carles M, Gispert R, Martinez-Alonso M, et al. (2010) The evolution of breast cancer mortality and the dissemination of mammography in Catalonia: an analysis by health region. Rev Esp Salud Publica 84: 691-703.
